# Genus-wide *Leptospira* core genome multilocus sequence typing for strain taxonomy and global surveillance

**DOI:** 10.1371/journal.pntd.0007374

**Published:** 2019-04-26

**Authors:** Julien Guglielmini, Pascale Bourhy, Olivier Schiettekatte, Farida Zinini, Sylvain Brisse, Mathieu Picardeau

**Affiliations:** 1 Institut Pasteur, Bioinformatics and Biostatistics Hub, C3BI, USR 3756 IP CNRS, Paris, France; 2 Institut Pasteur, Biology of Spirochetes unit, National Reference Center for Leptospirosis, Paris, France; 3 Université Paris Diderot, Ecole Doctorale BioSPC, Paris, France; 4 Institut Pasteur, Biodiversity and Epidemiology of Bacterial Pathogens, Paris, France; Baylor College of Medicine, UNITED STATES

## Abstract

*Leptospira* is a highly heterogeneous bacterial genus that can be divided into three evolutionary lineages and >300 serovars. The causative agents of leptospirosis are responsible of an emerging zoonotic disease worldwide. To advance our understanding of the biodiversity of *Leptospira* strains at the global level, we evaluated the performance of whole-genome sequencing (WGS) as a genus-wide strain classification and genotyping tool. Herein we propose a set of 545 highly conserved loci as a core genome MLST (cgMLST) genotyping scheme applicable to the entire *Leptospira* genus, including non-pathogenic species. Evaluation of cgMLST genotyping was undertaken with 509 genomes, including 327 newly sequenced genomes, from diverse species, sources and geographical locations. Phylogenetic analysis showed that cgMLST defines species, clades, subclades, clonal groups and cgMLST sequence types (cgST), with high precision and robustness to missing data. Novel *Leptospira* species, including a novel subclade named S2 (saprophytes 2), were identified. We defined clonal groups (CG) optimally using a single-linkage clustering threshold of 40 allelic mismatches. While some CGs such as *L*. *interrogans* CG6 (serogroup Icterohaemorrhagiae) are globally distributed, others are geographically restricted. cgMLST was congruent with classical MLST schemes, but had greatly improved resolution and broader applicability. Single nucleotide polymorphisms within single cgST groups was limited to <30 SNPs, underlining a potential role for cgMLST in epidemiological surveillance. Finally, cgMLST allowed identification of serogroups and closely related serovars. In conclusion, the proposed cgMLST strategy allows high-resolution genotyping of *Leptospira* isolates across the phylogenetic breadth of the genus. The unified genomic taxonomy of *Leptospira* strains, available publicly at http://bigsdb.pasteur.fr/leptospira, will facilitate global harmonization of *Leptospira* genotyping, strain emergence follow-up and novel collaborative studies of the epidemiology and evolution of this emerging pathogen.

## Author summary

Leptospirosis, caused by pathogenic *Leptospira* strains, is an emerging bacterial zoonotic disease mostly affecting humans in tropical countries. Despite its public health importance, little is known about the strains that are circulating worldwide due to the lack of a universal common language on strain types. In this work we describe a new strain genotyping and classification system that is highly standardized, thus facilitating global collaboration, and that can discriminate all members of the *Leptospira* genus at high resolution. We then examine the genetic diversity of *Leptospira* strains from different origins. This study provides a framework for optimizing diagnostic methods and epidemiological surveillance of leptospirosis.

## Introduction

Spirochetes constitute an evolutionarily and morphologically unique group of bacteria [1]. Pathogenic members of this phylum are the causative agents of several important diseases including leptospirosis, an emerging zoonotic disease with more than one million severe cases and 60,000 deaths every year worldwide, mostly in the tropical countries [2]. Pathogenic *Leptospira* species can cause a wide range of diseases in human, ranging from mild flu-like symptoms to severe complications, such as Weil's disease and pulmonary hemorrhagic syndrome, in which the case fatality rate can reach 40% [3]. Leptospirosis is expected to become more prominent worldwide due to climate change and the growing urban population living in slums. In addition, infections with pathogenic species can lead to major economic losses in livestock, as animal infections include *e*.*g*., abortion and loss of milk production [4].

The high public health and economic importance of *Leptospira* calls for better control of the infections the bacteria cause to both humans and animals. However, the control of *Leptospira* transmission is challenging for several reasons. First, the life cycle of pathogenic *Leptospira* is complex. Pathogenic leptospires are excreted through the urine of a wide range of animals including rodents which are asymptomatic reservoirs and livestock. Transmission to susceptible hosts usually occurs through contact with water contaminated with the urine of infected animals [5]. Therefore, multiple environmental sources of exposures, linked to multiple animal species, must be considered as possibilities.

Further complicated matters, the genus *Leptospira* is genetically highly heterogeneous and knowledge of its biodiversity remains largely incomplete. Taxonomically, the genus is currently subdivided into 35 species [6]. These species are ordered into three major evolutionary clades named according to their virulence status: pathogens, intermediates and saprophytes [1]. The agents of leptospirosis belong to two subclades, the pathogens (13 species) and the intermediates (11 species). The pathogenic species are responsible of the most severe infections in both human and animals, yet we know little about which component of the spirochete are critical for virulence. The species of the intermediates subclade are widely distributed in the environment [6–10] and they may be responsible for mild infections in both human and animals [11–19]. Intermediates possess most of the virulence factors found in the pathogens [1, 20]. In turn, the saprophytes form a single clade containing eleven species that are regarded as non-pathogenic environmental bacteria [1]. Saprophytes are relatively fast-growing *in vitro* when compared to the pathogens and lack the virulence factors described in infectious strains [1]. Classification into the three main clades has been typically performed using housekeeping and 16S rRNA genes sequencing [20].

Yet another barrier against leptospirosis control is the difficulty in isolating and cultivating *Leptospira*, which hinders optimal diagnostics of infections as well as laboratory identification, and hampers the constitution and maintenance of strain culture collections that are needed for microbiological studies and diagnostic or vaccine development purposes.

Finally, there is a lack of efficient strain typing methods that would allow tracking *Leptospira* strains (i) from their environmental or animal sources to their infected hosts and (ii) as they spread across time and space. Serotyping, which relies on the use of specific monoclonal antibodies, has led to the distinction of >300 serovars based on the structural heterogeneity of the surface-exposed lipopolysaccharides (LPS). This method has demonstrated an association of serovars with some animal reservoir hosts [21], even though the mechanisms that have allowed the adaptation of pathogenic *Leptospira* to various hosts are still unknown. However, serovar identification is currently performed by only two reference laboratories worldwide and is fastidious and time-consuming [22]. Furthermore, correlation between serotypes and genomic background is not always accurate, as the LPS biosynthetic locus (*rfb*) can be horizontally transferred between *Leptospira* species [23–25].

Molecular typing methods include pulsed-field gel electrophoresis (PFGE) [26, 27], and multilocus variable-number tandem-repeat analysis (MLVA) [28], but both methods have important practical limitations. Thus, PFGE [26] is not widely used and laborious, and only the most common serovars are typeable. More recently, multilocus sequence typing (MLST) was developed [29–31], but unfortunately three distinct MLST schemes have been proposed and applied to distinct collections of isolates, resulting in fragmentation of *Leptospira* epidemiological knowledge. Further, given the heterogeneity of *Leptospira*, the above methods are not universally applicable to all clades and species. In particular, MLST schemes are mainly focused on pathogens. As a consequence, current knowledge on the biodiversity and epidemiology of *Leptospira* is limited, and there is a critical need for a consensus *Leptospira* genotyping method that would be inclusive for its entire biodiversity, would facilitate fine-level strain discrimination for epidemiological purposes, and would reach high standardization allowing comparison of data from laboratories globally.

Whole-genome sequencing (WGS) has emerged as a powerful tool for bacterial strain classification and epidemiological typing [32]. The core genome MLST (cgMLST) approach, which extends the MLST concepts to the core genome, was demonstrated to be a useful high-resolution typing method in other bacterial species [33–36].

Taking advantage of the unique strain collection of the Reference Center for Leptospirosis in charge of the leptospirosis surveillance in mainland France and French overseas territories, our objectives were (i) First, to define based on genomic sequencing, the phylogenetic diversity of *Leptospira*, and its links with ecology and geography. In particular, our purpose was to shed light on the saprophyte and intermediate clusters, which have been scarcely studied thus far, and to include potentially novel species in this analysis. (ii) Second, we aimed to devise a genomic sequence-based genotyping method that is simultaneously universally applicable across the entire *Leptospira* genus and highly discriminatory at the strain level, and to propose a genomic taxonomy of *Leptospira* strains.

## Methods

### *Leptospira* strains

We sequenced 327 genomes from the collection of the National Reference Centre for Leptospirosis (Institut Pasteur, Paris, France), which is a globally representative strain collection of isolates from environmental, animal, and human samples gathered in the last 50 years. All strains and genome sequences used here are listed in S1 Table. *Leptospira* strains were grown at 30°C in liquid Ellinghausen, McCullough, Johnson and Harris (EMJH) medium. Species identification and serovar typing were performed at the National Reference Centre for Leptospirosis (Institut Pasteur, Paris, France) as previously described [37–39].

### Ethics statement

Collection of the strains was conducted according to the Declaration of Helsinki. A written informed consent from patients was not required as the study was conducted as part of routine surveillance of the national reference center and no additional clinical specimens were collected for the purpose of the study. Cultures originating from human samples were anonymized. Approval for bacterial isolation from soil and water was not required as the study was conducted as part of investigations into leptospirosis outbreaks. For New Caledonia, approval for bacterial isolation from the natural environment was obtained from the South Province (reference 1689–2017) and North Province (reference 60912-2002-2017).

### Whole-genome sequencing and assembly

Bacterial genomic DNA was purified using MagNA Pure 96 Instrument (Roche). Next-generation sequencing was performed by the Mutualized Platform for Microbiology (P2M) at Institut Pasteur, using the Nextera XT DNA Library Preparation kit (Illumina), the NextSeq 500 sequencing systems (Illumina), and the CLC Genomics Workbench 9 software (Qiagen) for analysis. Draft genomes with 50x minimum coverage, a total size < 5.3 Mb, and a minimum N50 of 10,000 nt were used for subsequent analysis. All raw reads generated and/ or contig sequences were submitted to NCBI under the project number PRJEB29877 and are available under genome accession numbers ERR3047203 to ERR3047514.

We also downloaded 182 assembled genome sequences from the NCBI and PATRIC (www.patricbrc.org) databases, including reference strains of previously described species [20] and representative isolates for each clade (S1 Table).

### cgMLST scheme definition, phylogenetic analysis and comparaison with MLST schemes

To determine a core gene set, 103 high-quality genome sequences of *Leptospira* covering the whole diversity of the *Leptospira* genus, *i*.*e*., representative isolates from the three clusters (50% from the pathogens, 12% from the intermediates, and 38% from the saprophytes) were selected (S1 Table); 50% of the genomes were downloaded from NCBI, the others were sequenced as described above.

From this set we inferred the genus core genome using the CoreGeneBuilder pipeline [40] and *L*. *interrogans* serovar Copenhageni strain Fiocruz L1-130 (GCF_000007685) as a reference. The pipeline’s first step relies on the eCAMBer software [41], which consists of a *de novo* annotation of the genomes (except the reference) using Prodigal [42] and the harmonization of the positions of the stop and start codons. In the next step, the core genome is inferred with a bidirectional best hits (BBH) approach as previously described by Touchon *et al*. [43]. We used CoreGeneBuilder default settings except for the synteny parameters (options–R and–S) both of which were set to 1. A gene was considered as part of the core genome if found in at least 90% of our genomes. Genes were not requested to be present in all genomes, as this stringent definition of a core genome would have resulted in too few genes given the diversity of *Leptospira*. Instead, the set of genes defined using the relaxed requirement of 90% presence can be viewed as a “soft core genome”. This resulted in an initial core genome containing 764 genes. We then filtered out some genes based on the following criteria. (i) First, we removed potential paralogs. Indeed, the presence of paralogs inside a typing scheme can lead to ambiguities, as a candidate gene might be attributable to two different core gene loci. To detect those potential paralogs, we compared each allele of each locus against all the alleles of all the other loci using the software BLAT [44]. If a single hit was found between two different loci (more than 70% protein identity between two alleles), we removed both. (ii) Second, we also removed genes that belong to one of the 3 existing *Leptospira* MLST schemes [29, 45, 46] and the ribosomal genes, so that they can be analyzed independently. (iii) Third, we also removed loci whose length varies too much among alleles, which is useful in reduceing ambiguities during the genotyping process. We aligned the protein sequences and removed those for which the alignment contained more than 10% of gaps (total number of gaps compared to the total number of characters). (iv) We removed loci containing ambiguous characters. (v) Finally, to avoid redundancy in the information contained within the cgMLST scheme, we removed loci that were overlapping in the reference genome using the definition of Prodigal [42]: a minimum of 60 bp of overlap if genes are on the same strand, and of 200 bp if genes are on different strands. The analysis resulted in the selection of 545 core genes listed in S2 Table and this cgMLST scheme was then used to analyze the presence of genes and to call alleles in 509 genomes (S1 Table), including the 103 genomes used for core genome definition. The allele and profiles definitions of the *Leptospira* cgMLST scheme were made publicly available through an Internet-accessible genotyping platform at https://bigsdb.pasteur.fr/leptospira/.

To derive a phylogenetic tree based on cgMLST gene loci, the allelic sequences of each locus were extracted and aligned as protein sequences using MAFFT v7 [47]. The concatenation of all loci yielded to a supermatrix of characters. IQ-TREE v1.5.4 [48] was used to infer a phylogenetic tree from this supermatrix of characters with an LG+G evolutionary model. Branch supports were assessed with both bootstrap (1,000 replicates) and aLRT-SH methods [49]. All trees were drawn using the iTOL webserver [50].

To evaluate classical MLST against the newly defined cgMLST scheme, all available *Leptospira* STs were downloaded from the Oxford University MLST database at https://pubmlst.org/leptospira/ [51] which comprises schemes 1, 2, and 3 developped by Boonsilp *et al*. [45], Varni *et al*. [46] and Ahmed *et al*. [29], respectively (S1 Table). MLST alleles derived from our WGS data were compared to the MLST database to determine the ST of our genome assemblies. Simpson index of discrimination and Wallace or Rand indices of concordance among partitions were computed using the web site http://www.comparingpartitions.info [52, 53].

## Results

### *Leptospira* isolates and genomes

A total of 327 *Leptospira* isolates were sequenced, covering the diversity of the *Leptospira* genus. A complementary set of 182 genome sequences of *Leptospira* strains, mostly reference strains from the *Leptospira* Genome project [20], was downloaded from GenBank and PATRIC (S1 Table). The total set of 509 genomes contained representatives of most *Leptospira* species currently described. The clusters of pathogens, intermediates and saprophytes were represented by 402, 31, and 76 genomes, respectively. Geographically, the dataset was highly diverse: strains were isolated from different geographical areas (Africa: 19, East Asia: 17, Caribbean: 13, Central America: 7, Europe: 73, Indian Ocean: 123, Middle East: 4, North America: 24, Oceania: 11, Pacific Ocean: 14, South America: 101, Southeast Asia: 97). The ecological sources of the strains were also diverse: 111 were from the environment, 226 were from humans, while the remaining isolates were from various animal hosts, such as rodents, cows, dogs, and pigs (S1 Table). The strains corresponded to 42 species including 15 novel species isolated from the environment in Japan, Mayotte, France, Malaysia, Algeria, and New Caledonia [54]. There were 26 serogroups and 73 serovars in the dataset (S1 Table). The strains selected for this study are therefore highly diverse geographically, ecologically and taxonomically.

The general features of the 509 genomes are reported in S1 Table and summarized in S1 Fig. Genomic assembly sizes ranged from 3,450,639 to 5,267,227 base pairs. Pathogens had a heterogeneous genome size, which was larger on average than the genome size of intermediates, which in turn had a larger genome than saprophytes (p < 0.001 for both comparisons). The genomic assemblies of pathogens were more fragmented (average contig number, 222) than those of the two other clusters (52 and 47 for the saprophytes and intermediates, respectively), which may reflect the high number of mobile elements in the pathogens [55]. The guanine+cytosine content (G+C%) of genomes was higher in the intermediates (42.39%) than in the saprophytes (38.27%, p < 1e-7) and in the pathogens (38.83%, p < 1e-7). Saprophytes were more homogeneous in their G+C% content than the two other clusters (S1 Fig).

### Genome-based phylogeny of the genus *Leptospira*

**Fig 1:**
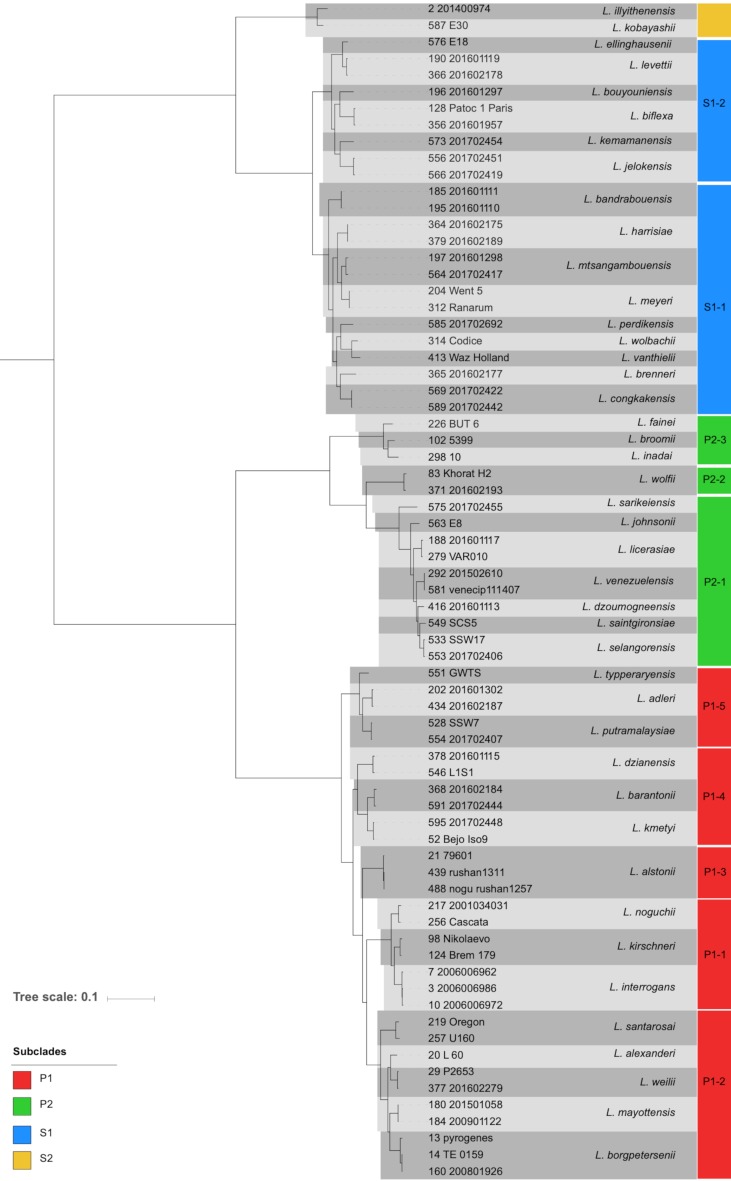
Phylogeny of representative strains of 42 *Leptospira* species. The phylogenetic tree was obtained from the concatenation of 545 amino-acid sequence alignments using IQ-TREE [48] and the maximum likelihood criterion. In addition to previously described *Leptospira* species, new species (S1 Table) from subclades S2 (*L*. *ilyithenensis* and *L*. *kobayashii*), S1 (*L*. *bouyouniensis*, *L*. *kemamanensis*, *L*. *jelokensis*, *L*. *bandrabouensis*, *L*. *mtsangambouensis*, *L*. *perdikensis*, *L*. *congkakensis*), P2 (*L*. *sarikeiensis*, *L*. *dzoumogneensis*, *L*. *selangorensis*), and P1 (*L*. *putramalaysiae* and *L*. *dzianensis*) are included. Species are grouped into subgroups (numbers within blocks in the column on the right) within each subclade (colors of blocks; see key).

To define the phylogenetic diversity of the dataset, 545 selected genes (see Methods, section cgMLST definition) were translated, aligned and then concatenated (S2 Table). The resulting phylogenetic tree is shown in S2 Fig. ANI analysis [54] revealed 42 species defined using the 95% ANI cutoff [56, 57], including 15 novel species for which a formal description was proposed elsewhere [54]. The phylogenetic tree with representatives of each species (Fig 1) is consistent with previous data [1] showing two major clades, the “saprophytes” containing species isolated in the natural environment and not responsible for infections and “pathogens” containing all the species responsible for infections in both humans and animals, plus environmental species for which the virulence status is not clearly established. This latter clade is further subdivided in two subclades that we named P1 (formerly described as the pathogen group) and P2 (formerly described as the intermediate group). Note that two strains previously assigned to the saprophytes (strains 201400974 and E30 isolated from the natural environment in Algeria and Japan, respectively) were clearly distinct from the other saprophytes and represent new species, named *L*. *ilyithenensis* and *L*. *kobayashii*, of a novel subclade within the clade of saprophytes. We named this new subclade S2 for convenience, in comparison to S1 which is constituted by species formerly described as the saprophyte group [54]. The basal position of the saprophyte clade with respect to P1 and P2 subclades is concordant with previous studies [58, 59]. The mean genetic distances among the three main subclades S1, P1 and P2 (S3 Fig) ranged between 0.33 substitutions per site (pathogens P1- intermediates P2) and 0.47 substitutions per site (intermediates P2- saprophytes S1), underlining the fact that these subclades are separated by large evolutionary distances. In contrast, mean intra-subclade genetic distances were 0.13 (saprophytes S1), 0.12 (pathogens P1) and 0.17 substitutions per site (intermediates P2), reflecting the higher heterogeneity and deeper phylogenetic branching of the intermediates P2 subclade. The distance between the new subclade S2 and saprophytes S1 was 0.29, showing that it lies close the P1-P2 inter-subclade distance.

**Fig 2:**
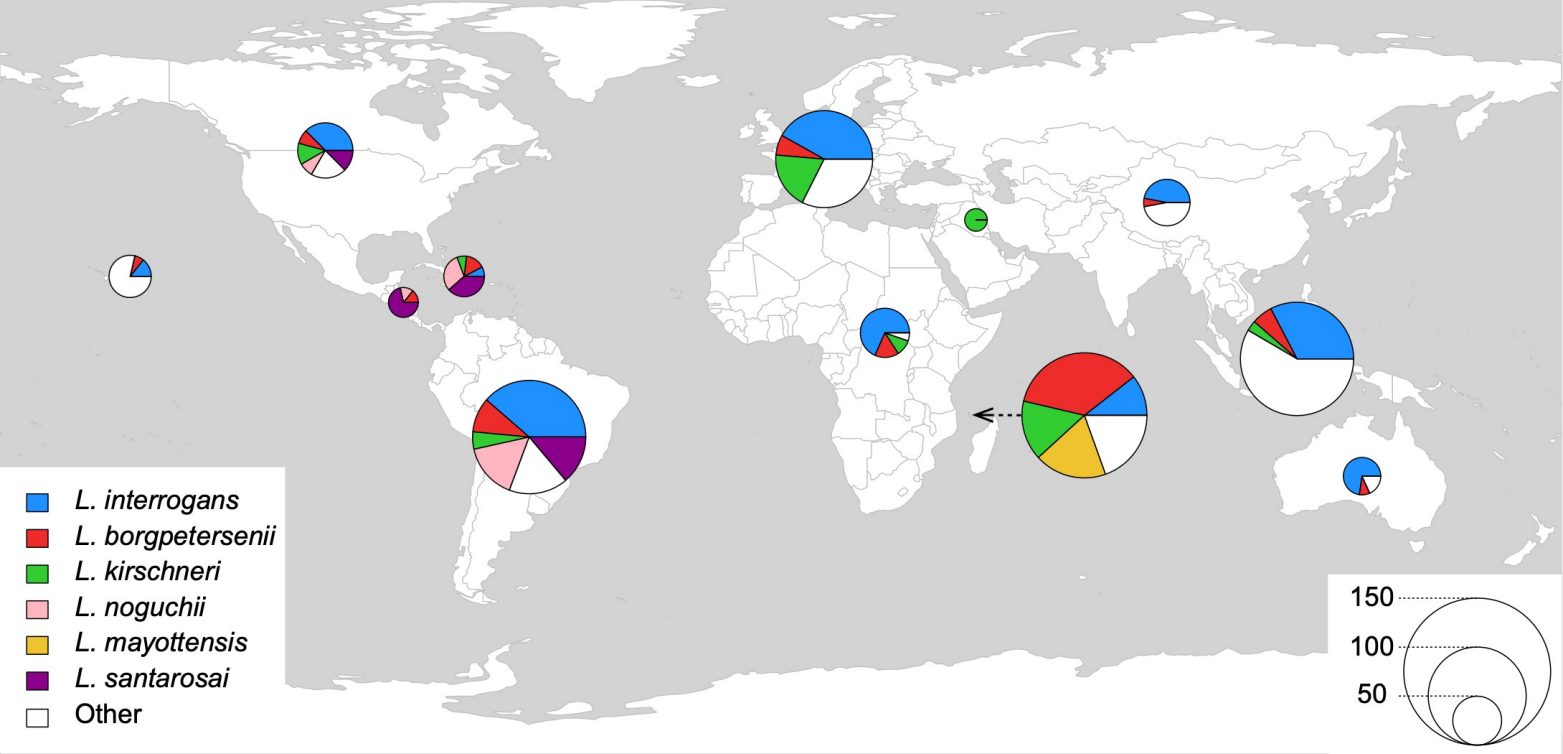
Geographic origins of the most frequent pathogenic *Leptospira* species. Each pie chart corresponds to a given world region. From left to right: Pacific Ocean, North America, Central America, Caribbean, South America, Europe, Africa, Middle East, Indian Ocean, Asia, Southeast Asia, Oceania. The size of the pie charts is proportional to the number of isolates (see key). The ‘Others’ category includes all pathogenic species not individualized in the key. The figure has been generated using R and the package "rworldmap", which is free under the GPL-2 and GPL-3 licenses (https://cran.r-project.org/web/packages/rworldmap/index.html).

We found that all species were monophyletic (S2 and S4 Fig). Furthermore, as expected, the intra-species distances were much lower than the inter-species. For example, *L*. *borgpetersenii* isolates formed a tight cluster with a maximum genetic divergence of 0.179 substitutions per site. Similarly, *L*. *interrogans* isolates showed high genetic relatedness, with a maximum distance of 0.033. This is remarkable given that both species are distributed worldwide (Fig 2). *L*. *mayottensis*, which is confined to the islands of Mayotte and Madagascar, showed a level of diversity of 0.008.

The phylogenetic analysis (Fig 1) revealed some structuration and led us to recognize several subgroups of species within subclades. Regarding the subclade P1, species *L*. *interrogans*, *L*. *noguchi* and *L*. *kirschneri* clustered into one subgroup (P1-1), whereas *L*. *borgpeterseni*, *L*. *alexanderi*, *L*. *weilii*, *L*. *mayottensis*, and *L*. *santarosai* formed a second subgroup (P1-2). Two other subgroups are constituted by *L*. *alstonii* (P1-3) and *L*. *kmetyi*, *L*. *barantonii* from New Caledonia [60] and *L*. *dzianensis* isolated from the environment in Mayotte (P1-4). Finally, subgroup P1-5 comprised *L*. *adleri*, *L*. *putramalaysiae* from the environment in Malaysia and *L*. *typperaryensis*. These subgroups are consistent with previous studies [20, 59, 61, 62].

To improve resolution, separate trees were constructed for the saprophytes S1 and the intermediates P2 (S4 Fig), showing the high level of genetic diversity among environmental isolates. The saprophytes were grouped into two subgroups. Subgroup 1 (S1-1) comprised *L*. *vanthielli*, *L*. *brenneri*, *L*. *wolbachii*; two new species: *L*. *perdikensis* and *L*. *congkakensis* from Malaysia; *L*. *meyeri*, *L*. *harrisiae* and the new species *L*. *mtsangambouenesis* and *L*. *bandrabouensis* isolated from Mayotte. Subgroup 2 (S1-2) comprised *L*. *biflexa* and three new species, *L*. *bouyouniensis*, *L*. *kemamanensis*, and *L*. *jelokensis*, isolated from Mayotte and Malaysia; and *L*. *levetti* and the new species *L*. *ellinghausenii* isolated from soil in Japan.

Among the intermediates P2, three subgroups were recognizable: subgroup P2-1 with *L*. *fainei*, *L*. *broomi*, *and L*. *inadai;* subgroup P2-2 with *L*. *wolffii;* and subgroup P2-3 with *L*. *venezuelensis*, *L*. *licerasiae*, *L*. *saintgironsiae* and four new species, named *L*. *dzoumogneensis*, *L*. *johnsonii*, *L*. *selangorensis*, and *L*. *sarikeiensis*, isolated from soils in Malaysia, Japan, and Mayotte (Figs 1, S2 and S4). A scheme for classifying *Leptospira* strains is proposed in S3 Table.

The phylogenetic structuration reflects a strong contrast between inter- and intra-species distances, which makes it possible to assign isolates at the species level based on their genome sequence-derived phylogenetic position. This led us to re-identify some isolates. For example, strain GWTS assigned to pathogen *L*. *alstonii* based on the 16S rRNA and *secY* genes [63, 64] did not cluster with the *L*. *alstonii* reference strain and formed a distinct branch in our phylogenetic tree (S2 Fig). Based on ANI values with representative species, including new species described in this study, it represents a new pathogenic species that we named *L*. *tipperaryensis* (S1 Table) [54]. Similarly, strains of serovar Rushan were previously identified as belonging to *L*. *noguchi* [65] but were phylogenetically clustered with *L*. *alstonii* (Figs 1 and S2) and had ANI values of 99.29% compared with the type strain of *L*. *alstonii*. These strains therefore appear to be new members of *L*. *alstonii*. Interestingly, the *L*. *alstonii* reference strain, of serovar Sichuan, was isolated from a frog [66], as were the strains from serovar Rushan, suggesting a tropism of this species for frogs.

### Geographical distribution of *Leptospira* species and *L*. *interrogans* sublineages

Species of the saprophytes and intermediates subclades were represented by few strains. In contrast, some species of pathogens subclade P1 were represented by multiple isolates (*e*.*g*., 160 for *L*. *interrogans*, 76 for *L*. *borgpetersenii*, 52 for *L*. *kirschneri*, 27 for *L*. *santarosai*, 27 for *L*. *noguchi* and 23 for *L*. *mayottensis*). Based on the present sample of *Leptospira* genomes, the geographic distribution of these species showed clear differences (Fig 2). *L*. *interrogans*, *L*. *borgpetersenii* and *L*. *kirschneri* were found in all world regions, even though *L*. *kirschneri* appeared more rarely in Asian and American samples than in Europe and Mayotte. In contrast, in our dataset, *L*. *santarosai* was only sampled from the American continent and the Caribbean islands and *L*. *noguchi* was found predominantly in the Americas and rarely in Asia. So far, *L*. *mayottensis* has been only isolated from the Indian Ocean islands (Fig 2).

We analyzed in more details the geographic distribution of the diversity of *L*. *interrogans*, the most common *Leptospira* species from human infections around the world, and which was the most represented in our dataset. S5 Fig presents a phylogenetic tree of the 152 *L*. *interrogans* isolates for which the geographic source was known; these were from 32 countries in all world regions. The data reveal extensive geographical spread of *L*. *interrogans* sublineages. Although some sublineages were sampled in a single world region (*e*.*g*., the sublineage containing serovars Szwajizak, Wewak, and Hawain originated in Oceania), it is clear that most sublineages are geographically widespread (S5 Fig). This is true even for genetically homogenous subgroups, which have limited phylogenetic depth and have therefore emerged recently. These data demonstrate the rapid spread of *L*. *interrogans* sublineages over large geographic distances.

### High-resolution cgMLST genotyping for *Leptospira* isolates

To develop a standardized subtyping strategy for *Leptospira*, we analyzed genome sequences using a gene-by-gene approach [34], based on the 545 genes that were highly conserved across the genus (S1 and S2 Tables). We define this set of gene loci as a core genome MLST (cgMLST) scheme [33, 34] for *Leptospira;* note that due to occasional absence of a few genes in some genomes, strictly speaking this set of genes is a ‘soft core genome’. The majority of cgMLST genes (527 loci per isolate on average, 96.7%) were called successfully (*i*.*e*., an allele was defined), including in the saprophytes S1 and intermediates P2 (S1 Table). The number of successfully called alleles per isolate ranged from 436 to 545 depending on the gene (S1 Table). Hence, this cgMLST scheme allows genotyping of all *Leptospira* genomes, with only a few missing data points.

For high-resolution subtyping, we defined cgMLST sequence types (cgST) as groups of cgMLST allelic profiles that are identical at all loci except for missing data, which are ignored in pairwise comparisons of allelic profiles (S1 Table). Considering the 509 genomes, there were 463 distinct profiles (defined by their cgST identifier, S1 Table), *i*.*e*., most genomes could be identified by a unique allelic profile. The discriminatory power of cgST classification (Simpson’s index) was 99.9%, much higher than that of MLST: for genomes that were typeable by cgMLST and the three MLST schemes [29, 45, 46], the Simpson indices of discrimination were 0.999 (confidence interval: 0.998–1.000), 0.793 (0.735–0.851), 0.787 (0.730–0.845) and 0.787 (0.730–0.845) for cgST, MLST1, MLST2 and MLST3, respectively. Hence, as expected, the use of 545 genes instead of 7 cgMLST largely improves our ability to distinguish among *Leptospira* isolates.

To assess the reproducibility and stability of cgST subtyping, sequencing replicates were performed for three isolates: *L*. *licerasiae* strain VAR010, *L*. *meyeri* strain Veldrat, and *L*. *interrogans* strain L495. The two replicates of the same isolate shared the same cgST, indicating high reproducibility of cgST classification. We next analyzed a culture‐attenuated strain of *L*. *interrogans* serovar Lai that had accumulated mutations (insertions, deletions, and single-nucleotide variations) in 101 genes after serial *in vitro* passages over several years [67]. The derived strain (cgST20) was clearly distinct from the virulent parental strain (cgST23, differing by 15 loci). Nevertheless, these subcultures were grouped together in the phylogenetic tree (S2 Fig). Similarly, a virulence-attenuated isolate of *L*. *interrogans* serovar Manilae passaged 67 times was sequenced [68] and compared with the corresponding parental virulent culture. The cgMLST analysis classified the 2 cultures as cgST31 and cgST32, differing by only 2 alleles out of 545 genes. These results illustrate the high resolutive power of cgMLST, which can distinguish genomes of isolates that evolved *in-vitro* over several generations.

To evaluate the genetic diversity among isolates classified into the same cgST (or groups of cgSTs differing only by missing data in some isolates), we analyzed the three most numerous ones (highlighted with colors in S1 Table, column cgST) using a whole-genome single nucleotide polymorphisms (SNP) approach. First, cgST128 and its related cgST123 and cgST308 comprised eight *L*. *borgpetersenii* isolates from Mayotte. These differed by a maximum of 16 SNPs, and five isolates had only up to 2 SNPs among themselves. Second, cgST262 and related cgSTs (cgST130, cgST321 and cgST396) comprised 11 isolates, also of *L*. *borgpetersenii* from Mayotte. These isolates differed among themselves by up to 23 SNPs. Finally, cgST482 and related cgST484 comprised seven *L*. *interrogans* isolates from cows in Uruguay; all these isolates were identical (no SNP) except one, which differed by only three SNPs from the others. These results show that isolates sharing the same cgST, or cgSTs that are identical except for missing data, are very closely related also based on whole-genome SNPs, and include levels of whole-genome SNPs that are compatible with the isolates being part of recent chains of transmissions [69, 70].

### A genomic taxonomy for *Leptospira* strains

To define groups of *Leptospira* strains based on cgMLST, we first explored the distribution of pairwise distances among all cgMLST profiles (S6 Fig). We also evaluated the quality of clustering, using the Silhouette index [71], resulting from the use of all possible threshold values (from 1 to 544) in single-linkage clustering (S7 Fig), revealing a plateau of maximal clustering quality between 40 and 300 allelic mismatches. Based on the above analyses, a threshold of 40 allelic differences was chosen as the cut-off value to define clonal groups (CG). In other words, a CG is defined as a group of cgMLST allelic profiles differing by no more than 40 allelic mismatches, out of 545 gene loci, from at least one other member of the group. This definition resulted in the identification of 237 CGs (S1 Table). To evaluate this choice as compared to alternative thresholds, we compared using the adjusted rand coefficient [72] the partitions (*i*.*e*., groups of isolates classified into the same CG) obtained using thresholds of 20, 30, 50, 60, 150, 200 and 300 mismatches (S7 Fig). Interestingly, confidence intervals overlapped with those of threshold 40 within a wide range of possible cutoff values (20 to 150). Hence, a choice of alternative thresholds in that range would have a limited impact on the resulting clusters. Finally, the effect of missing data (uncalled cgMLST alleles) on the clustering results was evaluated *in-silico* by introducing increasing amounts of missing data and assessing the resulting clusters of isolates as compared to their initial cluster (S8 Fig). This simulation showed that cluster assignment is robust to even high amounts of missing data (affecting up to 400 loci out of 545).

**Fig 3:**
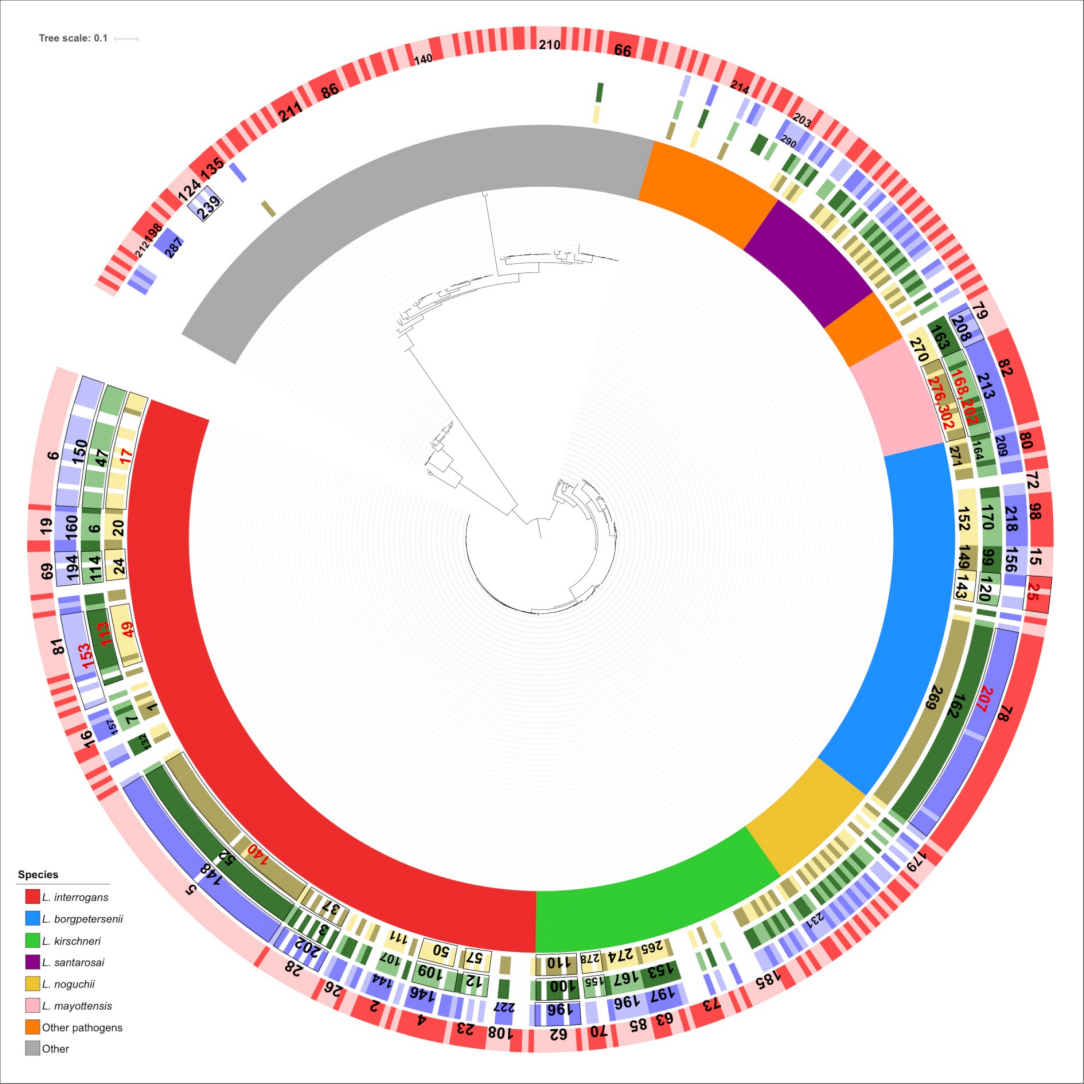
Comparison of cgMLST clustering with sequence types of the three classical MLST schemes currently in use. The inner colors overlaying the strain names represent the species (see colour key). The 4 outer circles represent ST and cgMLST clonal groups (CG) classifications (S1 Table), with alternating shades as the identifiers change along the circle. Yellow circle, scheme MLST1 [45]; green circle: scheme MLST2 [46]; blue circle: scheme MLST3 [29]. The red circle corresponds to the clonal group (CG) cgMLST classification. Clonal groups are defined as groups of allelic profiles differing by no more than 40 allelic mismatches out of 545 loci from any other member of the group. The numbers in the four circles correspond to the main STs/CGs (with more than 3 representative genomes) of the corresponding sector. Red numbers correspond to STs for which all genomes are part of the subclade but whose subclade also contain at least one member of another ST (i.e., paraphyletic STs). Red numbers separated by commas correspond to multiple main STs mixed together.

The clusters created at the 40-mismatch level represent a potentially useful genome-based taxonomy of *Leptospira* strains. To evaluate this classification system in comparison with previous *Leptospira* strain classifications, we first compared them to the 6- or 7-gene MLST classifications currently in use [29, 45, 46]. The three MLST classifications (S1 Table) were mapped onto the phylogenetic tree and their concordance with cgMLST was analyzed (Fig 3). A total of 260, 106, and 143 *Leptospira* STs are currently defined for MLST schemes 1, 2, and 3, respectively (April 2018; https://pubmlst.org/leptospira/). These MLST schemes were developed for strain typing of the main pathogenic *Leptospira* species but not for the saprophytes and intermediates [29, 30, 45, 46, 73]. As expected, saprophytes and most intermediates were not typeable by the three classical MLST schemes, whereas by design, all strains were typeable by cgMLST (Fig 3). Therefore, the typeability of the proposed cgMLST scheme appears greatly enhanced as compared with classical MLST.

We also assessed the concordance among assignments produced by the three MLST schemes and the cgMLST clustering into CGs, using Sankey diagrams (S9 Fig) and adjusted Rand and Wallace coefficients [72]. The adjusted Rand index of concordance of MLST with cgMLST was 0.86, 0.89 and 0.89 for MLST1 [45], MLST2 [46] and MLST3 [29], respectively. Wallace indices are not symmetrical, and thus produce two values: one for the comparison of MLST versus cgMLST clustering (*i*.*e*., how well MLST identity predicts CG identity), and one for the reciprocal comparison. The results were 0.86 and 0.86 for MLST1, 0.82 and 0.97 for MLST2, and 0.83 and 0.96 for MLST3. Hence, the CG accurately predicts with high accuracy the STs of MLST2 and MLST3. Only 4, 1 and 2 cgMLST clusters matched more than one MLST ST for scheme 1, 2 and 3, respectively (S9 Fig). Reciprocally, 26, 9 and 13 STs for MLST1, MLST2 and MLST3, respectively, were subdivided into more than one CG. In other words, despite accepting 40 mismatches within members of the groups, CG classification is still more discriminatory than each of the classical MLST systems. Note that although the low- and high-passage strains (see above) of *L*. *interrogans* serovar Lai and *L*. *interrogans* serovar Manilae were distinguishable at the level of cgST subtypes, they were classified into the same CG (CG16 and CG23, respectively), consistent with their recent evolutionary link.

To provide access to the cgMLST allele and profiles nomenclature, allowing for comparison and sharing of typing results among laboratories worldwide, a database was set-up and was made publicly accessible online (https://bigsdb.pasteur.fr/leptospira/). This database is based on the software framework Bacterial Isolate Genome Sequence Database (BIGSdb) [33, 34, 74].

### Phylogenetic distribution of serovars and correspondence with clonal groups

The distribution of serovars and serogroups along the phylogeny showed that most serogroups had a polyphyletic distribution. The fact that phylogenies can be in disagreement with serotyping was previously reported, and some serovars or related serovars from a same serogroup may belong to different species [21]. Thus, isolates from the same serogroup can be distributed in different species or sublineages within species. For example, *L*. *interrogans* strains of serogroup Australis or of serogroup Pyrogenes did not all cluster together in the phylogenetic tree (S2 Fig).

We investigated the correspondence of cgMLST groups with serovars. Serogroups (sg) were usually sub-divided into several CGs (S1 Table). For example, the 29 isolates of sg Australis were subdivided into 14 CGs, the 42 isolates of sg Grippotyphosa fell into 16 CGs, and the 20 isolates of sg Pyrogenes were grouped into 12 CGs. At the serovar level, highly related strains belonged to the same clonal group (S1 Table). This was the case for the 19 isolates from serovars Copenhageni and Icterohaemorrhagiae, which were clustered together in CG6, and for serovars Ratnupura and Vanderhoedeni (CG185, *L*. *kirschneri* sg Grippotyphosa) and Bajan and Barbudensis (CG179, *L*. *noguchii* sg Australis). However, some serovars were genetically more heterogeneous and were themselves sub-divided into different cgMLST clonal groups (e.g. *L*. *kirschneri* and *L*. *interrogans* sv Grippotyphosa: 6 CGs; *L*. *interrogans* sv Lai, 3 CGs; *L*. *interrogans* sv Pyrogenes: 8 CGs) (S1 Table). Therefore, cgMLST groups represent a useful classification system that is genome sequence-based and is complementary to serogroup and serovar classification, which are based on surface antigens.

### cgMLST and *Leptospira* epidemiology

To explore the links between cgMLST classifications and the epidemiology of *Leptospira* strains, we first analyzed the correspondence of cgMLST groups with hosts. It is well established that serovars are usually associated with a specific animal reservoir; for example, rats usually carry serovars of the Icterohaemorrhagiae serogroup; and serovar Canicola is associated with dogs [21]. Here, the most frequent cgMLST clonal groups of subclade P1 contained isolates obtained from both human and animals (except in Mayotte where few isolates have been isolated from animals). Thus, isolates of *L*. *interrogans* sg Pyrogenes (CG23), *L*. *borgpetersenii* sg Ballum (CG15) and *L*. *borgpetersenii* sg Javanica (CG25), associated with human leptospirosis, were clustered by cgMLST with rodent isolates, suggesting that these serogroups are maintained in rodents and that these animals represent reservoirs of human infections (S1 Table). Similarly, CG19 corresponding to serovar Sejroe comprised human and cattle isolates (S1 Table). Some CGs were found in an even larger range of hosts. For example, the 37 isolates belonging to CG5 (serovar Pomona) were obtained from humans, dogs and cows from seven countries. Likewise, CG28 contained isolates from dogs, rodents, pigs, and humans, indicating that some CGs or serotypes are not always restricted to specific hosts and may have a more generalist ecology. The environmental strains from our study were usually not grouped with animal or human isolates, as they formed distincts CGs.

We next analyzed 90 clinical isolates collected in the island of Mayotte (Indian Ocean) over a period of 10 years (2007–2017). cgMLST separated them into 10 CGs, which were highly congruent with their serotypes and species (S1 Table). Serogroup Mini was predominant (60%) and subdivided into five CGs, which agreed with their species assignments (CG63, CG83 and CG84 for *L*. *kirschneri*, CG78 for *L*. *borgpetersenii*, and CG79 for *L*. *mayottensis*). The most frequent CG was CG78, corresponding to 39 isolates, which were distributed into 25 cgSTs and were isolated over the 10-year period. Isolates belonging to *L*. *mayottensis* were sub-divided into two CGs, CG79 (n = 7, 5 cgSTs) and CG82 (n = 16, 14 cgSTs) (S1 Table). These two groups were previously recognized by PFGE, MLST and serotyping [37, 75]. Isolates from the island of Mayotte belonged to cgMLST groups that were not found in other world regions, consistent with the unique epidemiology of leptospirosis in this insular ecosystem [37, 39]. In contrast, multiple CGs were observed in different geographical locations around the world (S1 Table). The wide geographic distribution of CGs indicates that geographic spread of *Leptospira* strains is faster than their genetic evolution into distinct CGs.

We next analyzed the geographic distribution of the high-resolution cgMLST types (cgST). One of the most represented cgSTs (cgST482) in our dataset is constituted by *L*. *interrogans* serovar Pomona strains (n = 6) isolated from cattle in Uruguay [76] (S1 Table). Although five out of six of these strains have been isolated from the same farm and were undistinguishable by SNP analysis, one isolate from another region of the country differed from the group of five isolates by 3 SNPs. This shows that cgST classification could possibly inform on the epidemiological links among *Leptospira* isolates.

## Discussion

Until now, a consensus approach to characterize and compare *Leptospira* isolates has been lacking, limiting our understanding of the biology and epidemiology of strains within this important genus and impeding progress in establishing appropriate control and prevention measures. Advanced knowledge on the diversity and distribution of *Leptospira* strains is also essential for the design and evaluation of the efficacy of new vaccines and diagnostic tools.

This study lays a foundation for a comprehensive understanding of the biodiversity of *Leptospira* and for the epidemiological surveillance of medically important *Leptospira* pathogens. The availability of high-throughput sequencing technologies and the reduction of their costs makes genome sequencing a viable option as the new gold standard for *Leptospira* genotyping and taxonomy. Recently, 14 new species were identified based on genomic comparisons and a high degree of biodiversity of *Leptospira* species in soils and water was recently uncovered [6, 17, 75]. Besides, there is growing evidence that “intermediate” species are responsible for mild infections in humans [6, 8, 11–17, 19, 77, 78]. Novel genotyping methods should therefore encompass the entire genus, including both potentially pathogenic and non-pathogenic strains, in order to provide universal *Leptospira* strain characterization systems.

The classical MLST schemes were developped using six or seven genes with a focus on pathogenic *Leptospira* species [29, 45, 73]. More recently, a new MLST scheme was proposed and applied to a wider collection of strains, including a few intermediate species [46, 62]. However, none of these MLST methods enables the inclusion of all major *Leptospira* lineages, including saprophytic strains. Here we sought to develop a cgMLST strategy, which is an extension of conventional MLST at genome scale [34]. Our comparative genome analyses resulted in the identification of 764 genus-wide core genes, including 545 that were deemed suitable for use in cgMLST genotyping. This is in accordance with previous estimates of 700 to 1,000 *Leptopira* core genes [6, 20, 59]. Importantly, our cgMLST scheme was developed using genomes representing the entire breadth of the phylogenetic diversity of the genus and was validated using *Leptospira* strains from diverse sources and geographical locations.

The cgMLST scheme was used to construct amino-acid sequence-based phylogenetic trees that were consistent with previous work and current species designations. In addition, this work revealed the existence of novel *Leptospira* species isolated from soils and water across a wide geographic range (Algeria, Mayotte, Japan, New Caledonia and Malaysia), including species from the new subclade S2 that is phylogenetically related to the previously known saprophytes S1. This work confirms the high diversity of *Leptospira* species in the natural environment [6, 60], and the novel taxa were described more formally elsewhere [54]. Further, cgMLST-based phylogenetic analysis provides high-level resolution, allowing discrimination among closely related species and strains.

Much like classical MLST data, cgMLST data can be used to devise a classification of isolates using the single linkage algorithm [79]. Here we defined clonal groups based on cgMLST with a 40 allelic mismatches cut-off value. In order to optimize discrimination among groups, this threshold was chosen as the smallest threshold within the range of thresholds that maximized the quality of clustering. We demonstrated the robustness of CG classification to missing data and to threshold choice, and therefore propose that CG identifiers will become a practical and highly stable genomic taxonomy system for *Leptospira* strains. However, it must be underlined that clonal groups are broad classification categories that are of limited use for transmission studies, as illustrated by the wide geographical and temporal distribution of isolates from single clonal groups. Isolates belonging to the same clonal group always belonged to the same serogroup. Conversely, strains of a given serogroup can fall into phylogenetically unrelated clonal groups, suggesting that some *Leptospira* serogroups are derived from multiple independent ancestors. Further, strains belonging to the same serovar were not always clustered together by cgMLST, indicating that serovars can also be polyphyletic. In contrast, genetically related serovars were sometimes conflated by cgMLST clustering. These observations underline the complementarity of cgMLST clonal groups with previous classifications based on serotyping. cgMLST allows assigning *Leptospira* isolates both at the species and serogroup levels, and in most cases at the serovar level as well. With the increasing description of novel species and the continuous recording of strain diversity within species by surveillance networks and microbiology laboratories, a precise understanding of the biodiversity of *Leptospira* strains is needed. cgMLST might represent a useful standard for classification and nomenclature, and would advantageously replace the current classical MLST nomenclatures, which are incomplete, and the serotyping nomenclature, which is complex and does not always reflects phylogenetic relationships, as is the case for other pathogens [80].

Although many CGs were found in distinct geographic regions, the island of Mayotte was a notable exception in that its CGs were endemic. The lack of dissemination of CGs from Mayotte, or of colonization of Mayotte by cosmopolitan CGs such as those of Icterohaemorrhagiae, illustrates the unique ecosystem of this island [81]. However, whether the distribution of species or CGs reported here reflects strong endemicity, or is due to currently limited sampling, will be subject of future studies. As an example of our sampling limitations, *L*. *santarosai* is not only found in America as shown in Fig 2 but also in Taiwan where this species is the most frequently encountered species in patients [82]. Isolation of additional strains from both humans and animals will also be required to evaluate whether or not environmental strains belonging to subclades P1 and P2 have the ability to cause infections.

We propose a high-resolution classification of *Leptospira* strains into cgSTs, which correspond to groups of isolates with total sequence identity at the 545 cgMLST genes, with a tolerance of missing data. We showed that this level of discrimination is able to distinguish among *in-vitro* evolved cultures. Due to the occurrence of missing data, the cgMLST profiles of some isolates can match several distinct cgSTs. Isolates with identical cgST or belonging to groups of related cgSTs (defined as matching single isolates’ profiles) were shown to differ at the whole-genome scale by less than ~30 SNPs. This level of divergence is indicative that they share a very recent common ancestor and might be part of an ongoing transmission chain [69, 70], even though genomic epidemiology applications to *Leptospira* remain to be evaluated taking into account its specific mutation rate and transmission dynamics.

*L*. *interrogans* and *L*. *borgpetersenii* are ubiquitous pathogenic species. This is probably due to the fact that rodents are major reservoir hosts for these species [45]. Thus, *L*. *interrogans* strains belonging to serovars Copenhageni and Icterohaemorrhagiae share the same CG regardless of their geographic origin. This limited genetic diversity and broad geographic distribution (S5 Fig) is consistent with recent evolution/expansion following extensive migration of rodents, the main reservoir of serovars Copenhageni and Icterohaemorrhagiae, and multiple introductions due to modern global transport, in particular long-range, ship-based travel and trade. Due to this rapid geographic diffusion, little phylogeographic signal was present in the dataset, rendering challenging the reconstruction of the geographic origins of *L*. *interrogans* and its sublineages with confidence. By contrast, species such as *L*. *noguchi*, *L*. *kirschneri*, and *L*. *mayottensis* are not associated with rats and are largely confined in specific geographical areas. The pathogen *L*. *mayottensis* may have been introduced into Mayotte from Madagascar via the tenrec, a small terrestrial mammal [83].

This work provides a framework for the definition of *Leptospira* clades, subclades, subgroups, species, as well as strains at two levels of resolution (S3 Table). The possibility for laboratories around the world to identify the same strains using a unified nomenclature and a centralised genotyping database will facilitate the sharing and dissemination of knowledge on circulating *Leptospira* genotypes, worldwide. The cgMLST scheme will also enable early detection of new genotypes being introduced into locations where they are not usually found. The links between genotypes and their pathogenic potential and virulence will be an important subject for future studies. For yet unknown reasons, a limited number of *Leptospira* serovars are much more likely to cause severe disease than others [84–87]. The role of phages, plasmids, and horizontal transfer in the acquisition of virulence factors also remains to be determined. The molecular basis of host specificity is also largely unknown. Future dedicated studies will be needed to characterize the gene content of subclades, species and strains, and their association with the clinical presentation and outcome of *Leptospira* infections.

## Supporting information

S1 Table Summary of the *Leptospira* genomes used in this study. The characteristics of the 509 *Leptospira* isolates and their genomes are indicated; this set includes the 103 genomes representative of the diversity of *Leptospira* used for core genome definition (flagged with a value of 1 in the third column). Clonal groups (CG) based on single-linkage clustering of cgMLST allelic profiles with the 40 allelic differences threshold are indicated. cgSTs, *i*.*e*., profiles that differ by no allele other than for missing data, are given; some cgSTs are expressed as multiple numbers separated by semi-colons given the occurrence of missing data. The ST of the three MLST classifications, when defined, is indicated. The number of called alleles out of the 545 cgMLST loci is also provided.
(XLSX)

S2 Table Core genes selected for the cgMLST scheme. The genes are named after the corresponding locus tag in the reference strain (*L*. *interrogans* serovar Copenhageni str. Fiocruz L1-130).
(XLSX)

S3 Table Genomic classification levels proposed in this study for Leptospira strains. (XLSX)

S1 Fig Genome characteristics of *Leptospira* subclades. The variation of size, number of contigs and G+C% are indicated in each panel as the median, the first and third quartiles (hinges). The upper whisker extends from the hinge to the largest value no further than 1.5 * IQR from the hinge (where IQR is the inter-quartile range, or distance between the first and third quartiles). The lower whisker extends from the hinge to the smallest value at most 1.5 * IQR of the hinge. Data beyond the end of the whiskers are outliers.
(TIFF)

S2 Fig Maximum-likelihood phylogenetic tree of the 509 *Leptospira* isolates based on the concatenated alignment of 545 protein sequences. The tree was rooted on the branch separating the pathogens from the saprophyte and intermediates clusters. Three circles surround the tree; the colors of the internal circle represent the species; the colors of the middle circle correspond to the serogroups, and those of the outer circle to the host. Note that the two members of subclade S2 were placed on a long branch within the saprophyte subclade S1, unlike in Fig 1; this placement corresponds to a phylogenetic artefact.
(TIFF)

S3 Fig Distribution of the intra- and inter-subclade genetic distances. The distances were calculated from the concatenated alignment of the 545 cgMLST loci nucleotidic sequences of the 509 isolates used in this study, under the TN93 mode (https://doi.org/10.1093/oxfordjournals.molbev.a040023) and using the software goalign (https://github.com/fredericlemoine/goalign).
(TIFF)

S4 Fig Maximum-likelihood phylogenetic tree based on the concatenated alignment of the 545 cgMLST loci sequences of the isolates from the subclades S1 (panel 1) and P2 (panel B). Tip labels contain the name of the species when known. Species are grouped into subgroups within each subclade.
(TIFF)

S5 Fig Maximum-likelihood phylogenetic tree of 152 *L. interrogans* strains. The tree was built from the concatenated alignment of the 545 cgMLST protein sequences, and includes all *L*. *interrogans* strains for which the isolation location was known. The country of isolation and serogroup are indicated.
(TIFF)

S6 Fig Distribution of the number of cgMLST allelic differences between pairs of isolates among the 509 genomes. The inner panel corresponds to a zoom on the region between 0 and 100 allelic differences.
(TIFF)

S7 Fig Evolution of the silhouette index in relation to the number of mismatches used to cluster cgMLST profiles by single-linkage. For each possible mismatch treshold (1 to 544), we performed a single-linkage clustering of the cgMLST profiles and calculated the corresponding silhouette index. The better the clustering, the closest the index is to 1.
The upper panel represents different adjusted rand indices inferred using the "Comparing partitions" tool (http://www.comparingpartitions.info/?link=Tool) when comparing the clustering at the given mismatch threshold with the clustering at the 40 loci mismatch threshold. Vertical bars correspond to the 95% confidence interval.
(PDF)

S8 Fig Simulation of the impact of missing data on single-linkage clustering. For each profile (n = 359) belonging to a clonal group with at least 2 isolates, data were removed randomly so that the resulting profiles had from 25 to 525 missing alleles with a step size of 25; this was done 100 times for each profile and missing data point. The resulting profiles were then compared to all other (initial) isolates profiles, and all isolates that differed by less than the threshold (40 mismatches) were recorded. There are three possibilities. (1) If all recorded isolates belonged to the same CG, and the CG was the one of the test isolate, this is counted as a match (reproducible clustering). (2) If no isolate belonged to the CG of the test isolate, this is a mismatch. (3) If at least one isolate belonged to the cluster of the test isolate, and at least one other to another CG, this would lead through single-linkage clustering to the merging of initially separate CGs, and is counted as a fusion. We then computed the proportion of matches, mismatches and fusions in the simulation exercise. The figure presents the proportion of correct associations (matches) for each missing data level.
(TIFF)

S9 Fig Sankey diagram representing the correspondence between MLST and cgMLST clonal groups (CG) identifiers. The three panels correspond to each of the three classical MLST schemes, as indicated on top of the right columns. Connectors indicate the correspondence of ST and CG; the vertical width of connectors is related to the number of isolates with both identifiers; the numbers connected from left to right denote the CG (left) or the ST (right). Vertical order and colors are arbitrary and were automatically chosen by the software RAWGraphs (https://doi.org/10.1145/3125571.3125585).
(TIFF)

## References

[1] Picardeau M. Virulence of the zoonotic agent of leptospirosis: still terra incognita? Nat Rev Microbiol 2017;15:297–307. 10.1038/nrmicro.2017.5 28260786

[2] Costa F, Hagan JE, Calcagno J, Kane M, Torgerson P, Martinez-Silveira MS, et al Global Morbidity and Mortality of Leptospirosis: A Systematic Review. PLoS Negl Trop Dis 2015;9:e0003898 10.1371/journal.pntd.0003898 26379143 PMC4574773

[3] Haake DA, Levett PN. Leptospirosis in humans. Curr Top Microbiol Immunol. 2015;387:65–97. 10.1007/978-3-662-45059-8_5 25388133 PMC4442676

[4] Ellis WA. Animal Leptospirosis In: Adler B, editor. *Leptospira* and Leptospirosis: Springer; 2014 p. 99–137.

[5] Ko AI, Goarant C, Picardeau M. *Leptospira*: the dawn of the molecular genetics era for an emerging zoonotic pathogen. Nat Rev Microbiol 2009;7:736–47. 10.1038/nrmicro2208 19756012 PMC3384523

[6] Thibeaux R, Iraola G, Ferrés I, Bierque E, Girault D, Soupé-Gilbert ME, et al Deciphering the unexplored *Leptospira* diversity from soils uncovers genomic evolution to virulence. Microb Genom 2018;4. 10.1099/mgen.0.000144 PMC5857368 29310748

[7] Lall C, Kumar KV, Raj RV, Vedhagiri K, Vijayachari P. Prevalence and Diversity of Leptospires in Different Ecological Niches of Urban and Rural Areas of South Andaman Island. Microbes Environ 2016;31:79–82. 10.1264/jsme2.ME15149 26936796 PMC4791121

[8] Masuzawa T, Sakakibara K, Saito M, Hidaka Y, Villanueva SYAM, Yanagihara Y, et al Characterization of *Leptospira* species isolated from soil collected in Japan. Microbiol Immunol 2018;62:55–9. 10.1111/1348-0421.12551 29105847

[9] Chaiwattanarungruengpaisan S, Suwanpakdee S, Sangkachai N, Chamsai T, Taruyanon K, Thongdee M. Potential pathogenic *Leptospira* species isolated from waterfall in Thailand. Jpn J Infect Dis 2018;71:65–7. 10.7883/yoken.JJID.2017.363 29093324

[10] Thaipadungpanit J, Wuthiekanun V, Chantratita N, Yimsamran S, Amornchai P, Boonsilp S, et al *Leptospira* species in floodwater during the 2011 floods in the Bangkok Metropolitan Region, Thailand. Am J Trop Med Hyg. 2013;(89):794–6. 24002484 10.4269/ajtmh.13-0124 PMC3795115

[11] Tsuboi M, Koizumi N, Hayakawa K, Kanagawa S, Ohmagari N, Kato Y. Imported *Leptospira licerasiae* infection in traveler returning to Japan from Brazil. Emerg Infect Dis. 2017;23. 10.3201/eid2303.161262 PMC5382744 28221126

[12] Chiriboga J, Barragan VA, Arroyo G, Sosa A, Birdsell DN, España K, et al High Prevalence of Intermediate *Leptospira* spp. DNA in Febrile Humans from Urban and Rural Ecuador. Emerg Infect Dis. 2015;21:2141–7. 10.3201/eid2112.140659 26583534 PMC4672404

[13] Matthias MA, Ricaldi JN, Cespedes M, Diaz MM, Galloway RL, Saito M, et al Human leptospirosis caused by a new, antigenically unique leptospira associated with a Rattus species reservoir in the peruvian Amazon. PLoS Negl Trop Dis. 2008;2:e213 10.1371/journal.pntd.0000213 18382606 PMC2271056

[14] Slack AT, Kalambaheti T, Symonds ML, Dohnt MF, Galloway RL, Steigerwalt AG, et al *Leptospira wolffii* sp. nov., isolated from a human with suspected leptospirosis in Thailand. Int J Syst Evol Microbiol. 2008;58:2305–8. 10.1099/ijs.0.64947-0 18842846

[15] Levett PN, Morey RE, Galloway RL, Steigerwalt AG. *Leptospira broomii* sp. nov., isolated from humans with leptospirosis. Int J Syst Evol Microbiol. 2006;56:671–3. 10.1099/ijs.0.63783-0 16514048

[16] Schmid GP, Steere AC, Kornblatt AN, Kaufmann AF, Moss CW, Johnson RC, et al Newly recognized Leptospira species ("Leptospira inadai" serovar lyme) isolated from human skin. J Clin Microbiol 1986;24:484–6. 3760144 10.1128/jcm.24.3.484-486.1986 PMC268945

[17] Puche R, Ferrés I, Caraballo L, Rangel Y, Picardeau M, Takiff H, et al *Leptospira venezuelensis* sp. nov., a new member of the intermediate group isolated from rodents, cattle and humans. Int J Syst Evol Microbiol. 2018;68:513–7. 10.1099/ijsem.0.002528 29239713

[18] Balamurugan V, Gangadhar NL, Mohandoss N, Thirumalesh SR, Dhar M, Shome R, et al Characterization of Leptospira isolates from animals and humans: phylogenetic analysis identifies the prevalence of intermediate species in India. Springerplus. 2013;2:362 10.1186/2193-1801-2-362 23961424 PMC3736078

[19] Petersen AM, Boye K, Blom J, Schlichting P, Krogfelt KA. First isolation of *Leptospira fainei* serovar Hurstbridge from two human patients with Weil's syndrome. J Med Microbiol 2001;50:96–100. 10.1099/0022-1317-50-1-96 11192512

[20] Fouts DE, Matthias MA, Adhikarla H, Adler B, Berg DE, Bulach D, et al What Makes a Bacterial Species Pathogenic?: Comparative Genomic Analysis of the Genus Leptospira. PLoS Negl Trop Dis. 2016;10:e0004403 10.1371/journal.pntd.0004403 26890609 PMC4758666

[21] Levett PN. Leptospirosis. Clin Microbiol Rev. 2001;14:296–326. 10.1128/CMR.14.2.296-326.2001 11292640 PMC88975

[22] Hartskeerl RA, Smythe LD. The role of leptospirosis reference laboratories. Curr Top Microbiol Immunol. 2015;387:273–88. 10.1007/978-3-662-45059-8_11 25388139

[23] De la Peña-Moctezuma A, Bulach DM, Kalambaheti T, Adler B. Comparative analysis of the LPS biosynthetic loci of the genetic subtypes of serovar Hardjo: *Leptospira interrogans* subtype Hardjoprajitno and *Leptospira borgpetersenii* subtype Hardjobovis. FEMS Microbiol Lett 1999;177:319–26. 10.1111/j.1574-6968.1999.tb13749.x 10474199

[24] DelaPeña-Moctezuma A, Bulach DM, Kalambaheti T, Adler B. Genetic differences among the LPS biosynthetic loci of serovars of Leptospira interrogans and Leptospira borgpetersenii. FEMS Immunol Med. 2001;31:73–81. 10.1111/j.1574-695X.2001.tb01589.x 11476985

[25] Llanes A, Restrepo CM, Rajeev S. Whole Genome Sequencing Allows Better Understanding of the Evolutionary History of *Leptospira interrogans* Serovar Hardjo. PLos One. 2016;11:e0159387 10.1371/journal.pone.0159387 27442015 PMC4956267

[26] Galloway RL, Levett PN. Application and Validation of PFGE for Serovar Identification of Leptospira Clinical Isolates. PLos Neglected Tropical Diseases. 2010:e824 10.1371/journal.pntd.0000824 20856859 PMC2939049

[27] Herrmann JL, Bellenger E, Perolat P, Baranton G, Saint Girons I. Pulsed-field gel electrophoresis of *Not*I digests of leptospiral DNA: a new rapid method of serovar identification. J Clin Microbiol. 1992;30:1696–702. 1629323 10.1128/jcm.30.7.1696-1702.1992 PMC265366

[28] Salaün L, Mérien F, Gurianova S, Baranton G, Picardeau M. Application of multilocus variable-number tandem-repeat analysis for molecular typing of the agent of leptospirosis. J Clin Microbiol 2006;44:3954–62. 10.1128/JCM.00336-06 17088367 PMC1698352

[29] Ahmed N, Devi SM, Valverde Mde L, Vijayachari P, Machang'u RS, Ellis WA, et al Multilocus sequence typing method for identification and genotypic classification of pathogenic *Leptospira* species. Ann Clin Microbiol Antimicrob 2006;5:28 10.1186/1476-0711-5-28 17121682 PMC1664579

[30] Thaipadungpanit J, Wuthiekanun V, Chierakul W, Smythe LD, Petkanchanapong W, Limpaiboon R, et al A Dominant Clone of Leptospira interrogans Associated with an Outbreak of Human Leptospirosis in Thailand. PLoS Negl Trop Dis. 2007;1(1):e56 10.1371/journal.pntd.0000056 . 17989782 PMC2041815

[31] Leon A, Pronost S, Fortier G, Andre-Fontaine G, Leclercq R. Multilocus sequence analysis for typing *Leptospira interrogans* and *Leptospira kirschneri*. J Clin Microbiol 2010;48:581–5. 10.1128/JCM.00543-09 19955271 PMC2815645

[32] Schürch AC, Arredondo-Alonso S, Willems RJL, Goering RV. Whole genome sequencing options for bacterial strain typing and epidemiologic analysis based on single nucleotide polymorphism versus gene-by-gene-based approaches. Clin Microbiol Infect. 2018;24:350–4. 10.1016/j.cmi.2017.12.016 29309930

[33] Jolley KA, Maiden MC. BIGSdb: Scalable analysis of bacterial genome variation at the population level. BMC Bioinformatics. 2010;11:595 10.1186/1471-2105-11-595 21143983 PMC3004885

[34] Maiden MC, vanRensburg MJ, Bray JE, Earle SG, Ford SA, Jolley KA, et al MLST revisited: the gene-by-gene approach to bacterial genomics. Nat Rev Microbiol. 2013;11:728–36. 10.1038/nrmicro3093 23979428 PMC3980634

[35] Moura A, Criscuolo A, Pouseele H, Maury MM, Leclercq A, Tarr C, et al Whole genome-based population biology and epidemiological surveillance of *Listeria monocytogenes*. Nat Microbiol. 2016;2(16185). 10.1038/nmicrobiol.2016.185 PMC8903085 27723724

[36] Bialek-Davenet S, Criscuolo A, Ailloud F, Passet V, Jones L, Delannoy-Vieillard AS, et al Genomic definition of hypervirulent and multidrug-resistant *Klebsiella pneumoniae* clonal groups. Emerg Infect Dis. 2014;20:1812–20. 10.3201/eid2011.140206 25341126 PMC4214299

[37] Bourhy P, Collet L, Lernout T, Zinini F, Hartskeerl RA, van der Linden H, et al Human Leptospira isolates circulating in Mayotte (Indian Ocean) have unique serological and molecular feature. J Clin Microbiol. 2012;50:307–11. 10.1128/JCM.05931-11 22162544 PMC3264139

[38] Bourhy P, Herrmann-Storck C, Theodose R, Olive C, Nicolas M, Hochedez P, et al Serovar diversity of pathogenic Leptospira circulating in the French West Indies. PLoS Negl Trop Dis. 2013;7:e2114 10.1371/journal.pntd.0002114 23516654 PMC3597474

[39] Bourhy P, Collet L, Clément S, Huerre M, Ave P, Giry C, et al Isolation and characterization of new Leptospira genotypes from patients in Mayotte (Indian Ocean). PLoS Negl Trop Dis. 2010;4:e724 10.1371/journal.pntd.0000724 20582311 PMC2889827

[40] C3BI-pasteur-fr/CoreGeneBuilder (Version v1.0) [Internet]. 2016. Available from: 10.5281/zenodo.165206.

[41] Wozniak M, Wong L, Tiuryn J. eCAMBer: efficient support for large-scale comparative analysis of multiple bacterial strains. BMC Bioinformatics. 2014;15:65 10.1186/1471-2105-15-65 24597904 PMC4023553

[42] Hyatt D, Chen GL, Locascio PF, Land ML, Larimer FW, Hauser LJ. Prodigal: prokaryotic gene recognition and translation initiation site identification. BMC Bioinformatics. 2010;11:119 10.1186/1471-2105-11-119 20211023 PMC2848648

[43] Touchon M, Hoede C, Tenaillon O, Barbe V, Baeriswyl S, Bidet P, et al Organised genome dynamics in the *Escherichia coli* species results in highly diverse adaptive paths. PLoS Genet. 2009;1:e100034. 10.1371/journal.pgen.1000344 PMC2617782 19165319

[44] Kent WJ. BLAT—The BLAST-Like Alignment Tool. Genome Res. 2002;12:656–64. 10.1101/gr.229202 11932250 PMC187518

[45] Boonsilp S, Thaipadungpanit J, Amornchai P, Wuthiekanun V, Bailey MS, Holden MTG, et al A Single Multilocus Sequence Typing (MLST) Scheme for Seven Pathogenic Leptospira Species PLOS Negl Trop Dis. 2013. 10.1371/journal.pntd.0001954 PMC3554523 23359622

[46] Varni V, Ruybal P, Lauthier JJ, Tomasini N, Brihuega B, Koval A, et al Reassessment of MLST schemes for *Leptospira* spp. typing worldwide. Infect Genet Evol. 2014;22:216–22. 10.1016/j.meegid.2013.08.002 23932960

[47] Katoh K, Standley DM. MAFFT multiple sequence alignment software version 7: improvements in performance and usability. Mol Biol Evol. 2013;30:772–80. 10.1093/molbev/mst010 23329690 PMC3603318

[48] Nguyen LT, Schmidt HA, vonHaeseler A, Minh BQ. IQ-TREE: a fast and effective stochastic algorithm for estimating maximum-likelihood phylogenies. Mol Biol Evol 2015;32:268–74. 10.1093/molbev/msu300 25371430 PMC4271533

[49] Anisimova M, Gil M, Dufayard JF, Dessimoz C, Gascuel O. Survey of Branch Support Methods Demonstrates Accuracy, Power, and Robustness of Fast Likelihood-based Approximation Schemes. Systematic Biology. 2011;5:685–99. 10.1093/sysbio/syr041 PMC3158332 21540409

[50] Letunic I, Bork P. Interactive tree of life (iTOL) v3: an online tool for the display and annotation of phylogenetic and other trees. Nucleic Acids Res 2016;44:W242–5. 10.1093/nar/gkw290 27095192 PMC4987883

[51] Larsen MV, Cosentino S, Rasmussen S, Friis C, Hasman H, Marvig RL, et al Multilocus sequence typing of total-genome-sequenced bacteria. J Clin Microbiol 2012;50:1355–61. 10.1128/JCM.06094-11 22238442 PMC3318499

[52] Carrico JA, Pinto FR, Simas C, Nunes S, Sousa NG, Frazao N, et al Assessment of band-based similarity coefficients for automatic type and subtype classification of microbial isolates analyzed by pulsed-field gel electrophoresis. J Clin Microbiol. 2005;43:5483–90. 10.1128/JCM.43.11.5483-5490.2005 16272474 PMC1287802

[53] Severiano A, Pinto FR, Ramirez M, Carrico JA. Adjusted Wallace coefficient as a measure of congruence between typing methods. J Clin Microbiol. 2011;49:3997–4000. 10.1128/JCM.00624-11 21918028 PMC3209087

[54] Vincent AT, Schiettekatte S, Goarant C, Neela VK, Bernet E, Thibeaux R, et al Revisiting the taxonomy and evolution of pathogenicity of the genus *Leptospira* through the prism of genomics. PLoS Negl Trop Dis. 2019;in press. 10.1371/journal.pntd.0007270 PMC6532842 31120895

[55] Picardeau M. Genomics, Proteomics, and Genetics of Leptospira In: Adler B, editor. Leptospira and Leptospirosis: Springer-Verlag Berlin Heidelberg; 2015.

[56] Richter M, Rosselló-Móra R. Shifting the genomic gold standard for the prokaryotic species definition. Proc Natl Acad Sci USA. 2009;106:19126–31. 10.1073/pnas.0906412106 19855009 PMC2776425

[57] Goris J, Konstantinidis KT, Klappenbach JA, Coenye T, Vandamme P, Tiedje JM. DNA-DNA hybridization values and their relationship to whole-genome sequence similarities. Int J Syst Evol Microbiol 2007;57:81–91. 10.1099/ijs.0.64483-0 17220447

[58] Picardeau M, Bulach DM, Bouchier C, Zuerner RL, Zidane N, Wilson PJ, et al Genome sequence of the saprophyte *Leptospira biflexa* provides insights into the evolution of *Leptospira* and the pathogenesis of leptospirosis. PLoS ONE. 2008;3:e1607 10.1371/journal.pone.0001607 18270594 PMC2229662

[59] Xu Y, Zhu Y, Wang Y, Chang YF, Zhang Y, Jiang X, et al Whole genome sequencing revealed host adaptation-focused genomic plasticity of pathogenic *Leptospira*. Sci Rep. 2016;6:20020 10.1038/srep20020 26833181 PMC4735792

[60] Thibeaux R, Girault D, Bierque E, Soupé-Gilbert ME, Rettinger A, Douyère A, et al Biodiversity of Environmental Leptospira: Improving Identification and Revisiting the Diagnosis. Front Microbiol 2018;9:816 10.3389/fmicb.2018.00816 29765361 PMC5938396

[61] Nalam K, Ahmed A, Devi SM, Francalacci P, Baig M, Sechi LA, et al Genetic affinities within a large global collection of pathogenic Leptospira: implications for strain identification and molecular epidemiology. PLoS One. 2010;5:e12637 10.1371/journal.pone.0012637 20805987 PMC2929200

[62] Caimi K, Repetto SA, Varni V, Ruybal P. *Leptospira* species molecular epidemiology in the genomic era. Infect Genet Evol 2017;54:478–85. 10.1016/j.meegid.2017.08.013 28818623

[63] Nally JE, Arent Z, Bayles DO, Hornsby RL, Gilmore C, Regan S, et al Emerging Infectious Disease Implications of Invasive Mammalian Species: The Greater White-Toothed Shrew (Crocidura russula) Is Associated With a Novel Serovar of Pathogenic Leptospira in Ireland. PLoS Negl Trop Dis 2016;10:e0005174 10.1371/journal.pntd.0005174 27935961 PMC5147805

[64] Nally JE, Bayles DO, Hurley D, Fanning S, McMahon BJ, Arent Z. Complete Genome Sequence of Leptospira alstonii Serovar Room22 Strain GWTS #1. Genome Announc 2016;4:e01230–16. 10.1128/genomeA.01230-16 27834698 PMC5105091

[65] Brenner DJ, Kaufmann AF, Sulzer KR, Steigerwalt AG, Rogers FC, Weyant RS. Further determination of DNA relatedness between serogroups and serovars in the family *Leptospiraceae* witha proposal for *Leptospira alexanderi* sp. nov. and four new *Leptospira* genomospecies. Int J Syst Bacteriol. 1999;49:839–58. 10.1099/00207713-49-2-839 10319510

[66] Smythe L, Adler B, Hartskeerl RA, Galloway RL, Turenne CY, Levett PN. Classification of *Leptospira* genomospecies 1, genomospecies 3, genomospecies 4 and genomospecies 5 as *Leptospira alstonii* sp. nov., *Leptospira vanthielii* sp. nov., *Leptospira terpstrae* sp. nov., *Leptospira yanagawae* sp. nov., respectively. Int J Syst Evol Microbiol. 2012;63:1859–62. 10.1099/ijs.0.047324-0 22984140

[67] Zhong Y, Chang X, Cao XJ, Zhang Y, Zheng H, Zhu YZ, et al Comparative proteogenomic analysis of the *Leptospira interrogans* virulence-attenuated strain IPAV against the pathogenic strain 56601. Cell Res. 2011;21:1210–29. 10.1038/cr.2011.46 21423275 PMC3193473

[68] Satou K, Shimoji M, Tamotsu H, Juan A, Ashimine N, Shinzato M, et al Complete Genome Sequences of Low-Passage Virulent and High-Passage Avirulent Variants of Pathogenic Leptospira interrogans Serovar Manilae Strain UP-MMC-NIID, Originally Isolated from a Patient with Severe Leptospirosis, Determined Using PacBio Single-Molecule Real-Time Technology. Genome Announc 2015;3:e00882–15. 10.1128/genomeA.00882-15 26272567 PMC4536678

[69] Schürch AC, Arredondo-Alonso S, Willems RJL, Goering RV. Whole genome sequencing options for bacterial strain typing and epidemiologic analysis based on single nucleotide polymorphism versus gene-by-gene-based approaches. Clin Microbiol Infect. 2018; 24:350–354. 10.1016/j.cmi.2017.12.016 29309930

[70] Bentley SD, Parkhill J. Genomic perspectives on the evolution and spread of bacterial pathogens. Proc Biol Sci. 2015;282:20150488 10.1098/rspb.2015.0488 26702036 PMC4707741

[71] Rousseeuw PJ. Silhouettes: a Graphical Aid to the Interpretation and Validation of Cluster Analysis. Computational and Applied Mathematics. 1987;20:53–65.

[72] Carriço JA, Silva-Costa C, Melo-Cristino J, Pinto FR, deLencastre H, Almeida JS, et al Illustration of a common framework for relating multiple typing methods by application to macrolide-resistant *Streptococcus pyogenes*. J Clin Microbiol 2006;44:2524–32. 10.1128/JCM.02536-05 16825375 PMC1489512

[73] Ahmed A, Thaipadungpanit J, Boonsilp S, Wuthiekanun V, Nalam K, Spratt BG, et al Comparison of two multilocus sequence based genotyping schemes for Leptospira species. PLoS Negl Trop Dis 2011;5:e1374 10.1371/journal.pntd.0001374 22087342 PMC3210738

[74] Jolley KA, Bray JE, Maiden MCJ. Open-access bacterial population genomics: BIGSdb software, the PubMLST.org website and their applications. Wellcome Open Res. 2018;3:124 10.12688/wellcomeopenres.14826.1 30345391 PMC6192448

[75] Bourhy P, Collet L, Brisse S, Picardeau M. *Leptospira mayottensis* sp. nov., a pathogenic species of the genus *Leptospira* isolated from humans. Int J Syst Evol Microbiol. 2014;64:4061–7. 10.1099/ijs.0.066597-0 25249563 PMC4811635

[76] Zarantonelli L, Suanes A, Meny P, Buroni F, Nieves C, Salaberry X, et al Isolation of pathogenic Leptospira strains from naturally infected cattle in Uruguay reveals high serovar diversity, and uncovers a relevant risk for human leptospirosis. PLoS Negl Trop Dis. 2018;12:e0006694 10.1371/journal.pntd.0006694 30212451 PMC6136691

[77] Lehmann JS, Fouts DE, Haft DH, Cannella AP, Ricaldi JN, Brinkac L, et al Pathogenomic inference of virulence-associated genes in Leptospira interrogans. PLoS Negl Trop Dis. 2013;7:e2468 10.1371/journal.pntd.0002468 24098822 PMC3789758

[78] Ganoza CA, Matthias MA, Collins-Richards D, Brouwer KC, Cunningham CB, Segura ER, et al Determining risk for severe leptospirosis by molecular analysis of environmental surface waters for pathogenic Leptospira. PLoS Med. 2006;3:e308 10.1371/journal.pmed.0030308 16933963 PMC1551915

[79] Feil EJ. Small change: keeping pace with microevolution. Nat Rev Microbiol. 2004;2:483–95. 10.1038/nrmicro904 15152204

[80] Achtman M, Wain J, Weill FX, Nair S, Zhou Z, Sangal V, et al Multilocus sequence typing as a replacement for serotyping in Salmonella enterica. PLoS Pathog. 2012;8:e1002776 10.1371/journal.ppat.1002776 22737074 PMC3380943

[81] Dietrich M, Gomard Y, Lagadec E, Ramasindrazana B, LeMinter G, Guernier V, et al Biogeography of *Leptospira* in wild animal communities inhabiting the insular ecosystem of the western Indian Ocean islands and neighboring Africa. Emerg Microbes Infect. 2018;7:57 10.1038/s41426-018-0059-4 29615623 PMC5883017

[82] Wang HK, Lee MH, Chen YC, Hsueh PR, Chang SC. Factors associated with severity and mortality in patients with confirmed leptospirosis at a regional hospital in northern Taiwan. J Microbiol Immunol Infect. 2018;S1684–118:30161–0. 10.1016/j.jmii.2018.05.005 29934034

[83] Lagadec E, Gomard Y, Le Minter G, Cordonin C, Cardinale E, Ramasindrazana B, et al Identification of Tenrec ecaudatus, a Wild Mammal Introduced to Mayotte Island, as a Reservoir of the Newly Identified Human Pathogenic Leptospira mayottensis. PLoS Negl Trop Dis 2016;10:e0004933 10.1371/journal.pntd.0004933 27574792 PMC5004980

[84] Tubiana S, Mikulski M, Becam J, Lacassin F, Lefèvre P, Gourinat AC, et al Risk factors and predictors of severe leptospirosis in New Caledonia. PLoS Negl Trop Dis 2013;7:e1991 10.1371/journal.pntd.0001991 23326614 PMC3542117

[85] Hochedez P, Theodose R, Olive C, Bourhy P, Hurtrel G, Vignier N, et al Factors Associated with Severe Leptospirosis, Martinique, 2010–2013. Emerg Infect Dis 2015;21:2221–4. 10.3201/eid2112.141099 26583702 PMC4672444

[86] Taylor AJ, Paris DH, Newton PN. A Systematic Review of the Mortality from Untreated Leptospirosis. PLoS Negl Trop Dis. 2015;9:e0003866 10.1371/journal.pntd.0003866 26110270 PMC4482028

[87] Goldstein RE, Lin RC, Langston CE, Scrivani PV, Erb HN, Barr SC. Influence of infecting serogroup on clinical features of leptospirosis in dogs. J Vet Intern Med 2006;20:489–94. 16734079 10.1892/0891-6640(2006)20[489:ioisoc]2.0.co;2

