# Correction: Genus-wide *Leptospira* core genome multilocus sequence typing for strain taxonomy and global surveillance

**DOI:** 10.1371/journal.pntd.0008673

**Published:** 2020-08-21

**Authors:** Julien Guglielmini, Pascale Bourhy, Olivier Schiettekatte, Farida Zinini, Sylvain Brisse, Mathieu Picardeau

The Funding section listed in the article [1] is incomplete and the contribution of the Pasteur International Joint Research Unit "Integrative Microbiology of Zoonotic Agents" (IMiZA) was mistakenly omitted. The updated Funding section reads:

“This work was supported in part by a donation from Foundation MSD to the ‘PIBnet’ programme of Institut Pasteur, by Public Health France (SPF), by Institut Pasteur through grant PTR 30–2017, and the Pasteur International Joint Research Unit "Integrative Microbiology of Zoonotic Agents" (IMiZA). This work was part of the PhD thesis of O. S. who received financial support from “Université Paris Diderot” and “Sorbonne Paris Cité”. The funders had no role in the design, conduct or conclusions of the study.”

In the Acknowledgments section the sharing of *Leptospira* genomes by contributors was mistakenly omitted. The updated Acknowledgments section reads:

“We thank the technicians of the National Reference Center for Leptospirosis, in particular Céline Lorioux, for cultures and typing of Leptospira strains, and Evelyne Bégaud and Chantal Bizet from Collection de l'Institut Pasteur (CIP) for providing type and reference strains. We thank Cyrille Goarant (Institut Pasteur de Nouméa, New Caledonia), Anissa Amara Korba (Institut Pasteur d’Alger, Algeria), Fairuz Amran (Institute for Medical Research, Malaysia), Toshiyuki Masuzawa (Chiba Institute of Science, Japan), Vasantha Kumari Neela (Universiti Putra Malaysia, Malaysia) and Alejandro Buschiazzo, Leticia Zarantonelli and the consortium "Grupo de Trabajo Interinstitucional de Leptospirosis” (Institut Pasteur Montevideo/UdelaR/INIA/MGAP, Uruguay) for sharing *Leptospira* genomes or isolates. We also thank Vincent Enouf and the team of core facility P2M (Institut Pasteur, Mutualized Platform for Microbiology) for genomic sequencing.”
