# Supplementary figures and images for: Genus-wide Leptospira core genome multilocus sequence typing for strain taxonomy and global surveillance
Source: PLoS Negl Trop Dis. 2019 Apr 26;13(4):e0007374. doi: 10.1371/journal.pntd.0007374 (PMC6513109; doi:10.1371/journal.pntd.0007374)

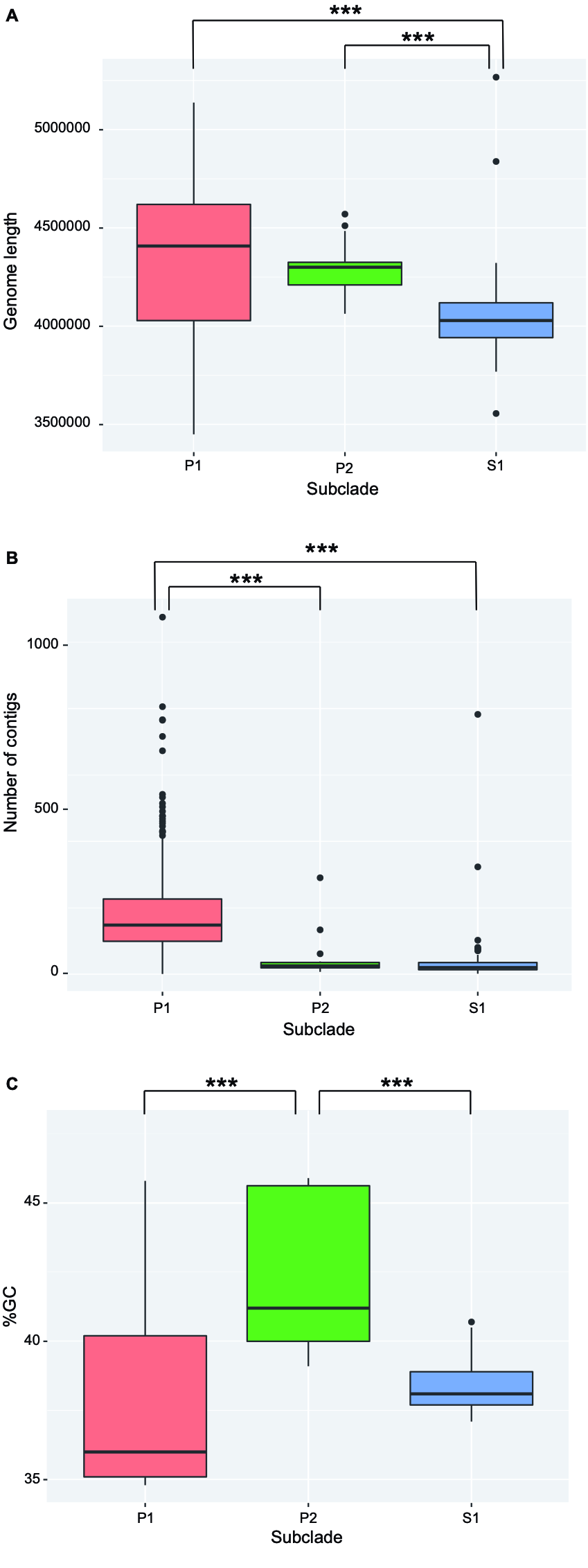

Supplement: S1 Fig — The variation of size, number of contigs and G+C% are indicated in each panel as the median, the first and third quartiles (hinges). The upper whisker extends from the hinge to the largest value no further than 1.5 * IQR from the hinge (where IQR is the inter-quartile range, or distance between the first and third quartiles). The lower whisker extends from the hinge to the smallest value at most 1.5 * IQR of the hinge. Data beyond the end of the whiskers are outliers. (TIFF) [file pntd.0007374.s004.tiff]

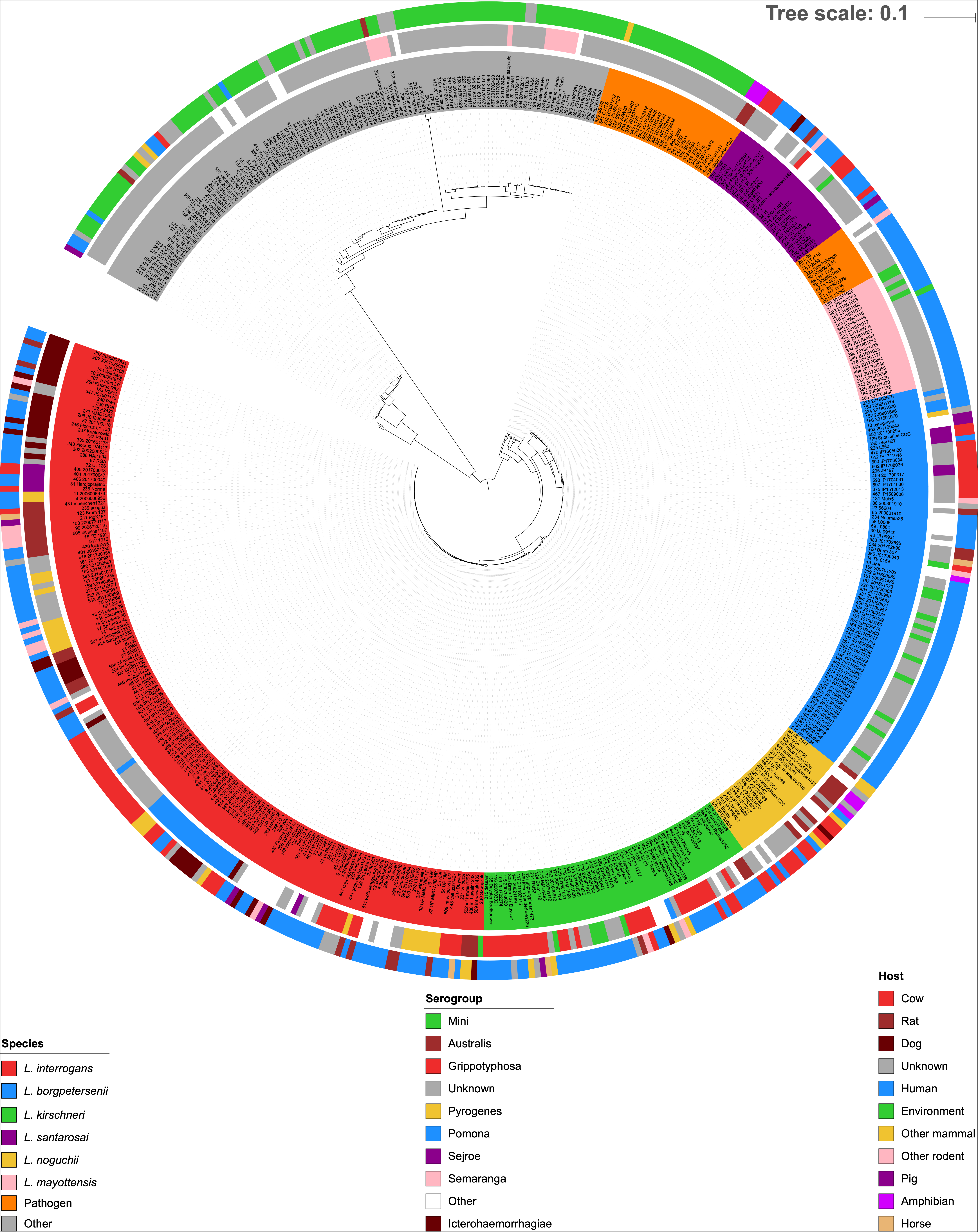

Supplement: S2 Fig — The tree was rooted on the branch separating the pathogens from the saprophyte and intermediates clusters. Three circles surround the tree; the colors of the internal circle represent the species; the colors of the middle circle correspond to the serogroups, and those of the outer circle to the host. Note that the two members of subclade S2 were placed on a long branch within the saprophyte subclade S1, unlike in Fig 1; this placement corresponds to a phylogenetic artefact. (TIFF) [file pntd.0007374.s005.tiff]

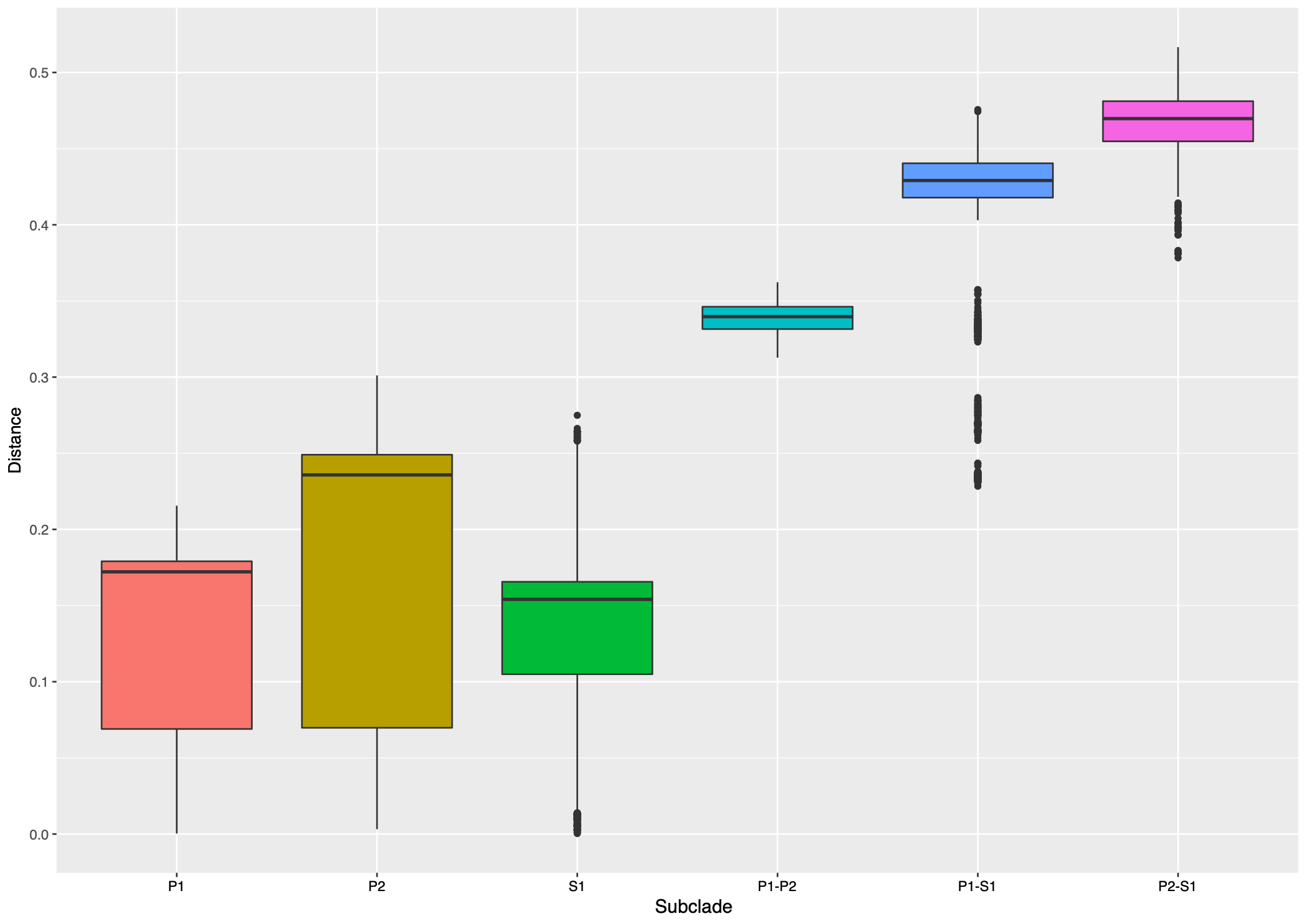

Supplement: S3 Fig — The distances were calculated from the concatenated alignment of the 545 cgMLST loci nucleotidic sequences of the 509 isolates used in this study, under the TN93 mode (https://doi.org/10.1093/oxfordjournals.molbev.a040023) and using the software goalign (https://github.com/fredericlemoine/goalign). (TIFF) [file pntd.0007374.s006.tiff]

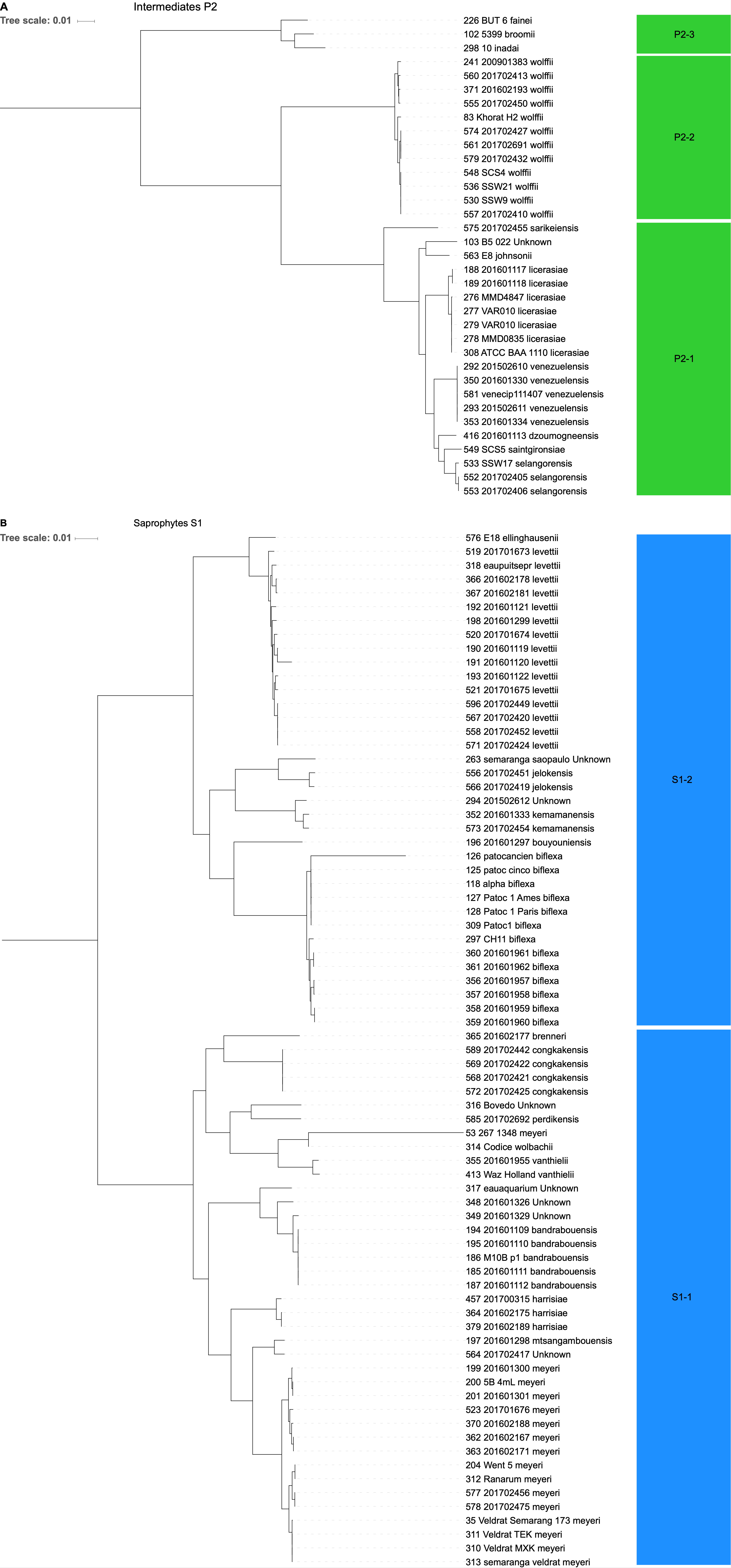

Supplement: S4 Fig — Tip labels contain the name of the species when known. Species are grouped into subgroups within each subclade. (TIFF) [file pntd.0007374.s007.tiff]

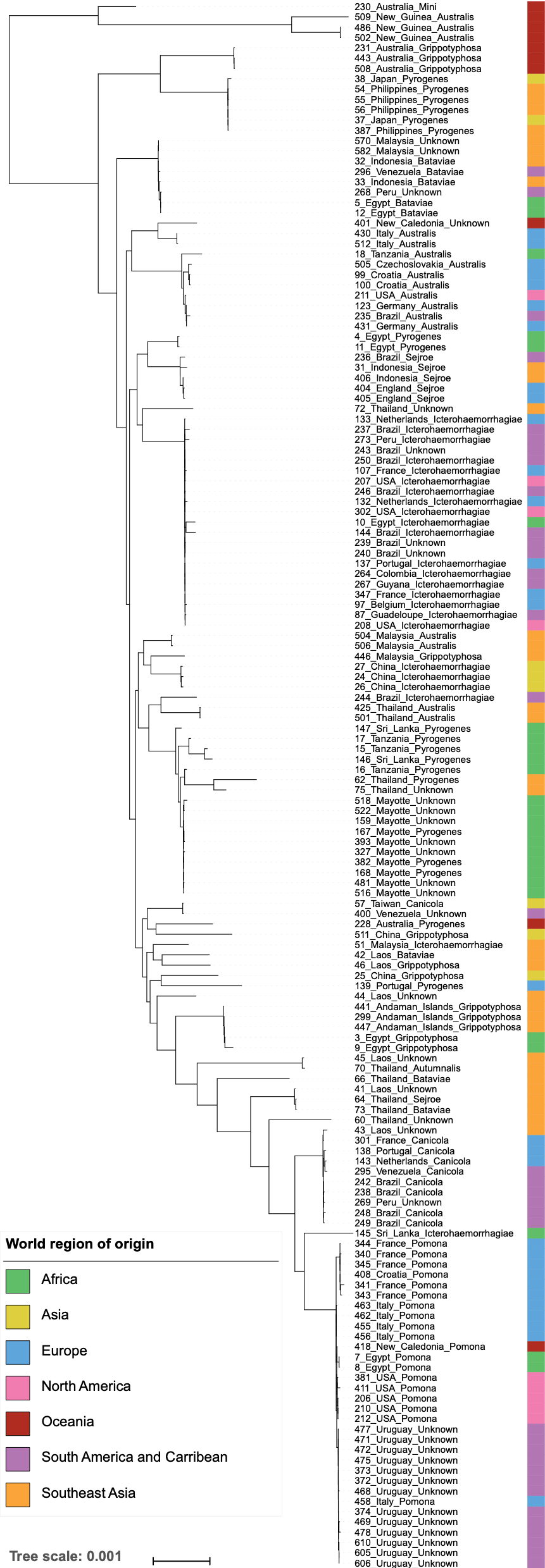

Supplement: S5 Fig — The tree was built from the concatenated alignment of the 545 cgMLST protein sequences, and includes all L. interrogans strains for which the isolation location was known. The country of isolation and serogroup are indicated. (TIFF) [file pntd.0007374.s008.tiff]

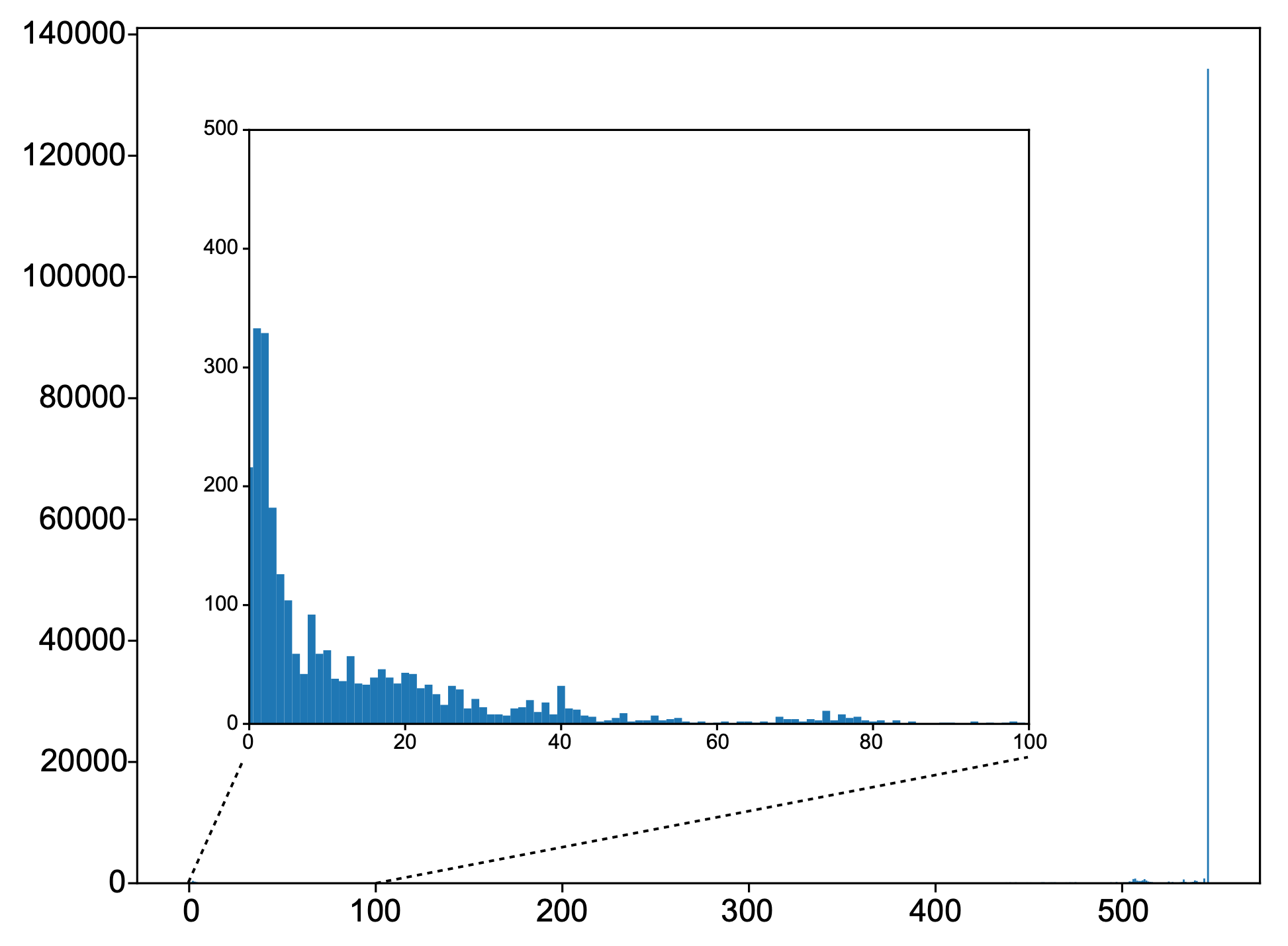

Supplement: S6 Fig — The inner panel corresponds to a zoom on the region between 0 and 100 allelic differences. (TIFF) [file pntd.0007374.s009.tiff]

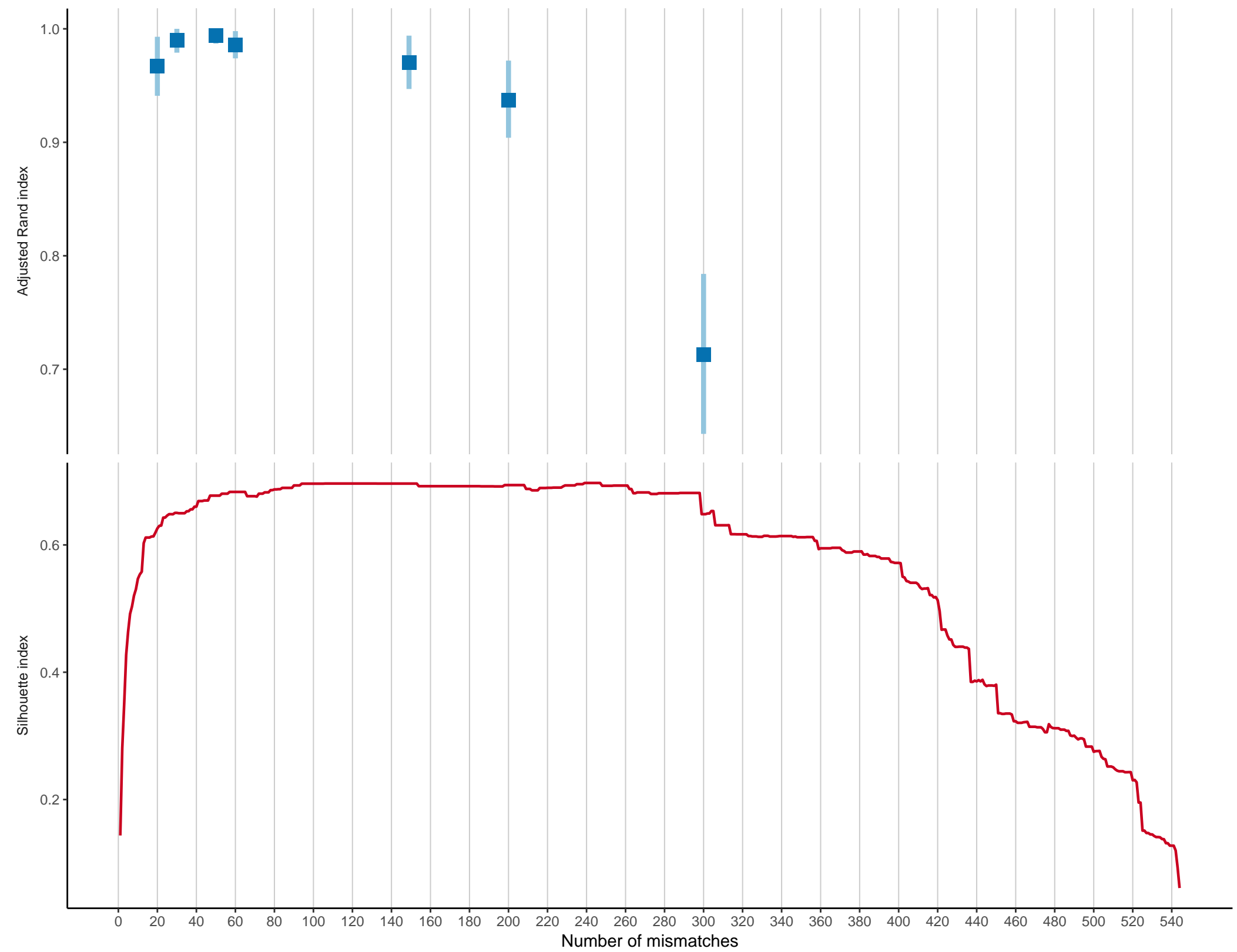

Supplement: S7 Fig — For each possible mismatch treshold (1 to 544), we performed a single-linkage clustering of the cgMLST profiles and calculated the corresponding silhouette index. The better the clustering, the closest the index is to 1. The upper panel represents different adjusted rand indices inferred using the "Comparing partitions" tool (http://www.comparingpartitions.info/?link=Tool) when comparing the clustering at the given mismatch threshold with the clustering at the 40 loci mismatch threshold. Vertical bars correspond to the 95% confidence interval. (PDF) [file pntd.0007374.s010.pdf]

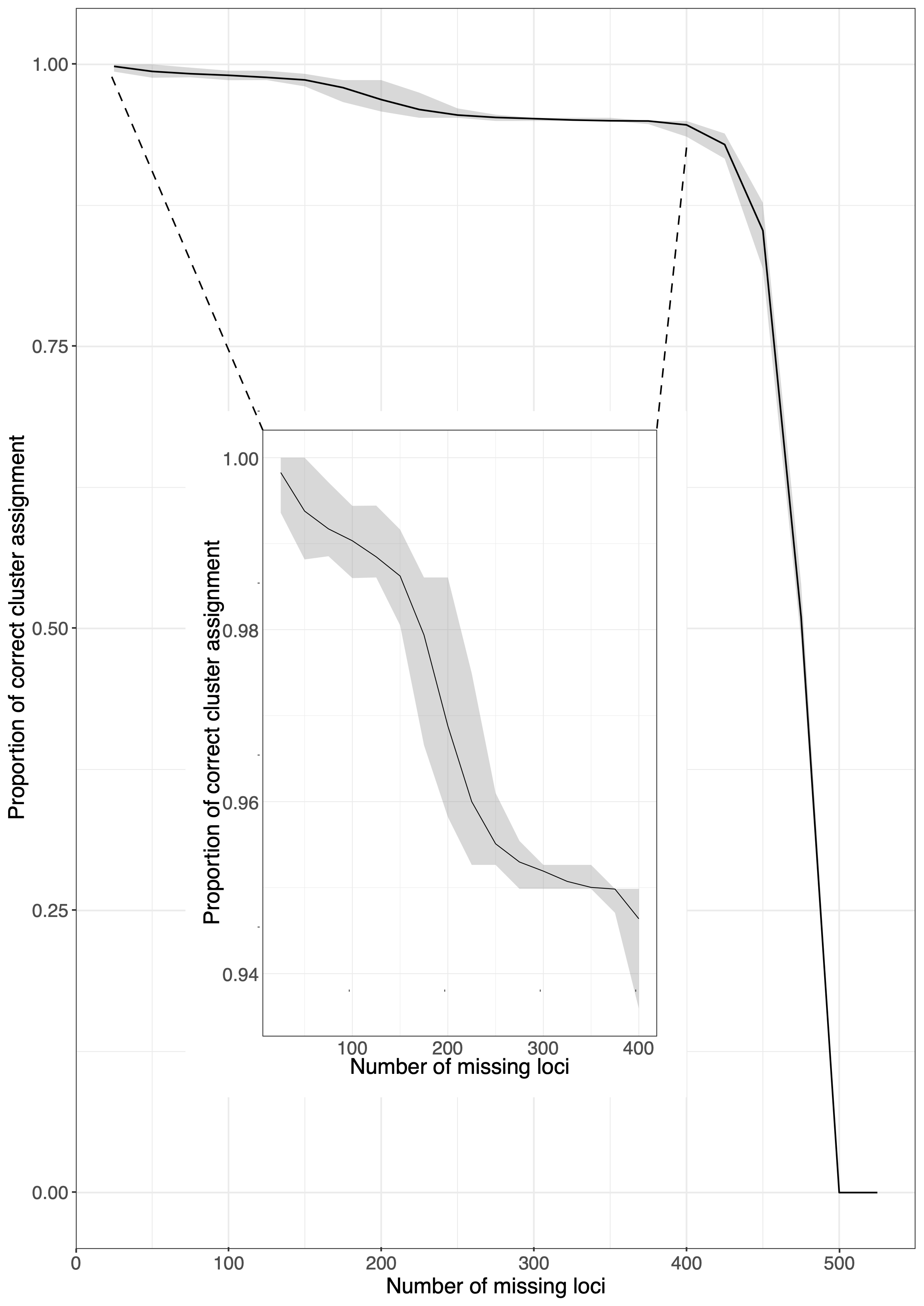

Supplement: S8 Fig — For each profile (n = 359) belonging to a clonal group with at least 2 isolates, data were removed randomly so that the resulting profiles had from 25 to 525 missing alleles with a step size of 25; this was done 100 times for each profile and missing data point. The resulting profiles were then compared to all other (initial) isolates profiles, and all isolates that differed by less than the threshold (40 mismatches) were recorded. There are three possibilities. (1) If all recorded isolates belonged to the same CG, and the CG was the one of the test isolate, this is counted as a match (reproducible clustering). (2) If no isolate belonged to the CG of the test isolate, this is a mismatch. (3) If at least one isolate belonged to the cluster of the test isolate, and at least one other to another CG, this would lead through single-linkage clustering to the merging of initially separate CGs, and is counted as a fusion. We then computed the proportion of matches, mismatches and fusions in the simulation exercise. The figure presents the proportion of correct associations (matches) for each missing data level. (TIFF) [file pntd.0007374.s011.tiff]
